# Walnut (*Juglans regia* L.) Volatile Compounds Indicate Kernel and Oil Oxidation

**DOI:** 10.3390/foods10020329

**Published:** 2021-02-04

**Authors:** Filipa S. Grilo, Selina C. Wang

**Affiliations:** 1 Department of Food Science and Technology, University of California, Davis, CA 95616, USA; fsgrilo@ucdavis.edu; 2 Olive Center, Robert Mondavi Institute for Wine and Food Science, University of California, Davis, CA 95616, USA

**Keywords:** peroxide value, oxidative stability, tocopherols, phenols, shelf-life, principal components analysis, hierarchical cluster analysis

## Abstract

Kernel oxidation susceptibility and pellicle darkening are among the biggest concerns regarding walnut quality. Monitoring oxidation is crucial to preserve quality from production to consumption. Chemical oxidation parameters (peroxide value and UV absorbances), fatty acid profile, tocopherols, phenols, and volatiles in ‘Chandler’ and ‘Howard’ kernels were studied at different time points during 28 weeks of storage to evaluate potential oxidation markers. During storage, peroxide value, UV absorbances, and volatiles concentration increased; oxidative stability, phenols, and tocopherols decreased, while fatty acid profile was unaffected. ‘Chandler’ had a lower peroxide value, K_232_, and K_268_; and higher kernel and oil oxidative stability compared to ‘Howard’. Phenols and tocopherols decreased 1.2-fold in ‘Chandler’ and 1.3-fold in ‘Howard’. Using multivariate analysis, samples were discriminated in three groups according with their oxidative levels. Increases of volatiles in oil and kernel were associated with higher oxidative levels. Pentanal, 2-methylpropanal, hexanal, *(E)*-2-pentenal, 3-octanone, octanal, *(Z)*-2-penten-1-ol, hexanol, *(E)*-2-octenal, 1-octen-3-ol, benzaldehyde, *(E,E)*-2,4-nonadienal, and hexanoic acid in kernels were adequate at distinguishing oxidation levels and as oxidative markers in walnuts. Kernel volatiles is a useful measurement for walnut oxidation during storage without any prior fat extraction.

## 1. Introduction

Walnut (*Juglans regia* L.) belongs to the Juglandaceae family that originated in Persia, with production and consumption spread throughout the world [1]. Nowadays, walnuts are grown in Asia, United States, Europe, North Africa, and South America. China and the United States represent more than 60% of walnut production worldwide, with the United States being the biggest exporter [2]. Both cultivars used in this study, ‘Chandler’ and ‘Howard’, were introduced by the University of California, Davis, walnut breeding program in 1984. ‘Howard’ is characterized by higher yields and disposition to become rancid compared to ‘Chandler’. ‘Chandler’ has extra-light kernel color and has become a reference in kernel quality for future breeding programs in California (McGranahan and Leslie, 2006).

Walnuts have generated great interest in recent years due to their nutritional value [3]. The beneficial effects of walnut consumption are well recognized and include protection from cardiovascular diseases, diabetes, and reduction of total cholesterol and LDL cholesterol [4]. Additionally, fatty acid composition, particularly unsaturated fatty acids, has an important role in the health benefit of walnuts. However, unsaturated fatty acids such as oleic acid, linoleic, and linolenic acids are highly susceptible to a free-radical chain reaction and start to breakdown in the presence of oxygen and high temperatures [5,6]. The non-enzymatic autoxidation of unsaturated fatty acids with oxygen forms hydroperoxides which eventually break down into volatile compounds, giving the perception of rancidity [7,8,9]. Thus, high unsaturated fatty acids result in low stability and short shelf life of walnut products due to rancidity development from oxidation and lipolysis. Since walnuts are subjected to minimal processing, few postharvest opportunities exist to reduce oxidative rancidity, which are mainly cultivar and proper handling during harvesting, drying, and storage [4]. 

Oxidative rancidity in nuts is commonly measured by peroxide value (PV), free fatty acidity (FFA), or sensory [10,11]. However, PV and FFA can only be measured in oil after extraction from nuts and do not always change linearly through oxidation stages and storage [11]. Sensory evaluations can be inconsistent, and training of panelists can be time-consuming and expensive. In industry, the use of induction time (i.e., Rancimat) for oxidative stability is widely used to measure oxidative rancidity on the extracted oil of high-fat content foods [11]. Even though oxidative deterioration in nuts can be evaluated by analytical methods or sensory, it is crucial to select the appropriate oxidative indicators and methodologies to distinguish samples at different oxidation levels. Kernels are a complex matrix where antioxidants and other bioactive compounds’ interaction with water or protein content influence their oxidative stability [4,12,13]. Currently, there is little information on the relations between chemical changes of kernels and oxidation measurements of the oil extracted from kernels; nor is there a comparison of chemical composition between ‘Chandler’ and ‘Howard’ cultivars, especially during storage. The objective of this work was to understand which chemical compounds present in the kernel and oil contribute the most for the oxidative stability in the walnuts and which chemical measurements can be used as oxidation indicators during storage. This study also provides information about the oxidation susceptibility of the two most common cultivars planted in United States and can be used as a resource for postharvest handling and storage best practices and future breeding programs.

## 2. Materials and Methods

### 2.1. Material

Walnut fruits (*Juglans regia*—‘Chandler’ and ‘Howard’) were harvested at full maturity (onset of hull split) from commercial plantations at Wheatland, California, USA. Without the hull, the fruits were dried at 30 ± 2 °C in a day’s time using a commercial air dryer and then unshelled mechanically.

### 2.2. Chemicals

HPLC grade methanol, HPLC grade hexane, and reagent grade ethanol were purchased from Fisher Scientific (Fairlawn, NJ, USA). γ-tocopherol, δ-tocopherol, α-tocopherol, α-tocopherol acetate, and refined olive oil were purchased from Sigma-Aldrich (St. Louis, MO, USA). Volatile standards were purchased from Sigma-Aldrich (St. Louis, MO). The water was obtained from a Milli-Q-Plus water purification system (Millipore, Milford, MA, USA).

### 2.3. Walnut Storage

Walnuts (18 kg) were stored in four (4.5 kg of kernels each) open transparent containers (65 cm × 50 cm × 30 cm) in temperature-controlled chambers (25 ± 2 °C) at 50% RH exposed to fluorescent light for seven months. Samples of 1.5 kg of kernels were collected for oil extraction and analysis every week during the first month of storage and every four weeks for the following six months. Kernels (1 kg) were sampled every week during the first four weeks of the study and every four weeks for the following six months of storage for a totality of 11 sampling points. Oil was extracted within 3 h after each sampling.

### 2.4. Walnut Oil Extraction

Walnut kernels were conditioned to reach 3% (*w*/*w*) of moisture content, sprinkled with fresh water, to ensure uniformity of extraction conditions within samples [14]. Samples were kept in a vacuum bag at 5 °C until the desired moisture was reached. Pressing was carried out at 27 °C by using an electrical resistance-heating ring attached around the press barrel. A screw press, KK oil Prince F universal (Reut, Germany), with a 7 mm restriction die and a screw speed of 20 rpm was used to extract the oil. The machine run for 10 min without kernels to reach the desired temperature for extraction. On each sampling point three separate oil extraction were performed from three different batches of walnut kernels.

### 2.5. Moisture Content

Walnuts samples (40.0± 0.1 g) were grounded using a Cuisinart Mini-Prep Plus® food processor (East Windsor, NJ, USA), model DLC-2A., weighed in a 600 mL beaker and dried in the oven at 105 °C until constant weight. After the sample reached room temperature in a desiccator the weight of the dry paste was registered. Each sample was analyzed in triplicates.

### 2.6. Fat Content

The total fat content was obtained by Soxhlet standard extraction mode of the Buchi extraction system (E800, Buchi Labortechnik AG, Flawil, Switzerland) using the previously dried ground walnuts with n-hexane for 50 extraction cycles and 30 minutes rinsing

### 2.7. Quality Parameters

Standard methods of American Oil Chemistry Society [15] were used to determine free acidity (Ca 5a-40 (09)), peroxide value (Cd 8b-90(09)), and UV absorbances (K_232_, K_268_) (Ch 5-91(09)). Each treatment was analyzed in triplicates.

### 2.8. Oxidative Stability

A Rancimat apparatus 617 (Metrohm AG, Herisau, Switzerland) was used to measure oxidative stability. Oil samples (3 g) and ground kernels (0.5 g) were weighed into test tubes and oxidized at 110 °C with 20 l/h of airflow. Each treatment was analyzed in triplicates.

### 2.9. Kernel Color Score

Individual kernel color was evaluated following the Dried Fruit Association (DFA) guidelines [16]. DFA is based on a chart for color evaluation that classified kernels into one of the four categories: ‘Extra light’ (1), ‘light’ (2), ‘light amber’ (3), and ‘amber’ (4). The same person consistently evaluated the kernel during the experiment. Forty kernels from each treatment were evaluated.

### 2.10. Fatty Acid Profile

The fatty acid composition of oil samples was determined as methyl esters by gas chromatography/mass spectrometry according to Tapia et al. [17]. Aliquots of 0.1 g of sample were diluted in 1ml of n-hexane and agitated for 10 sec. Subsequently, 0.1 mL of a 2N KOH solution in methanol was added and mixed in a vortex for 2 min. After the solution turned clear and transparent, 500 µL of the upper phase, containing the fatty acid methyl esters, was decanted, and diluted with n-hexane to a final volume of 1 mL and analyzed within 12 h from preparation. Samples were analyzed using a gas chromatograph (7890A, Agilent Technologies, Palo Alto, CA, USA) equipped with a split injector and a flame ionization detector. A ZB-23 capillary column (20 m, 180 µm, 0.2 µm) was used. An initial column temperature of 80 °C was used for 0.5 min, then programmed to 175 °C at the rate of 65 °C/min, and finally to 230 °C at the rate of 7 °C/min. At each stage of programming, the temperature was held for 0, 0.5, and 5 min, respectively. The injector and detector were held at 250 °C and 260 °C, respectively. A sample of 1 µL was injected. Peak areas of 10 fatty acids and their quantification were performed using Agilent open Lab ChemStation for Windows. Identification of fatty acid methyl esters was carried out using a mix of 37-component fatty acid methyl esters purchased from Supelco (Sigma-Aldrich, St. Louis, MO, USA). Each sample was analyzed in triplicates. Individual fatty acids were expressed as the percentage of total fatty acids.

### 2.11. Tocopherols Extraction and Analysis

Extraction was performed according to Gimeno et al. [18] with some modifications. Oil (40 µl) was briefly vortexed in 160 µL of hexane. 600 µL of methanol and 200 µL of internal standard solution (α-tocopherol acetate in ethanol, 300 µg/mL) were added. The sample was vortexed for 1 min and centrifuged (1788.8× *g* force, 5 min, Beckman GS-15R). Samples were stored at −20 °C to allow the separation between oil and organic phase. The organic extract was filtered (0.45 µm, nylon) and analyzed using UPLC-DAD. A blank followed by a standard mix (δ-tocopherol, γ-tocopherol, and α-tocopherol) was run after every fifteen samples. The analysis was performed on an Agilent 1290 Infinity II LC system with a diode-array detector using an Agilent ZORBAX Eclipse Plus C18 column (3.5 µm, 3 × 100 mm). The mobile phase was methanol:water (96:4) with 20 µL injection volume and flow rate of 1.0 mL/min. The total run time was 12 min with DAD signal recorded at 292 nm. Tocopherols were identified by their retention time in comparison with the standards. Each sample was analyzed in triplicates. 

### 2.12. Total Phenols in Kernel and Oil

Phenols from kernel and oil were extracted using a method adapted from conditions described in previous studies [19]. A double extraction of 0.4 g of ground kernel in n-hexane (6 + 4 mL) are vortexed for 2 min followed by 5 min ultrasound and centrifuged at 2000× *g* for 10 min. Then, 0.4 g of the previously combined extract (for kernel analysis) and 1 g of oil (for oil analysis) are extracted using 20 mL in two extraction (10 + 10 mL) of MeOH:H2O:HCOOH (80:20:0.1), with 2 min vortex followed by 5 min ultrasound and centrifugation at 2000× *g* for 10 min. The supernatant was collected, and the solid residues were re-extracted a second time as described above. The extracts were combined and membrane-filtered through cellulose filters (0.45 μm pore size; Macherey-Nagel, Düren, Germany). Total phenolic compounds from the extracts above were quantified by the Folin−Ciocalteu method [20] using a calibration curve of gallic acid and expressed as gram per kilogram of kernel or oil. Each sample was analyzed in triplicates.

### 2.13. Volatile Compounds in Kernel and Oil

Solid phase microextraction (SPME) was used to extract walnut volatile compounds. Sample of oil or ground kernel (3.0 ± 0.1 g), with 4-methyl-2-pentanol as internal standard (2.5 mg/kg), was analysed in a 20 mL glass vial and with a PTFE/silicone septum (Agilent Technologies, Palo Alto, CA). After 10 min equilibration time at 40 °C, a solid-phase microextraction (SPME) fiber (DVB/CAR/PDMS, Sigma-Aldrich, St. Louis, MO) was exposed to the sample headspace for 40 min. The volatile compounds were analysed using a GC system (Agilent Technologies) that comprise an autosampler (Agilent PAL RSI 85) with 45 positions, a gas chromatograph (GC Agilent 7820A), and a mass spectrometer (Agilent 5977B) with an electron impact source and a quadrupole analyzer. Compounds were separated using a Supelcowax 10 (30 m × 0.25 mm × 0.25 μm, Sigma-Aldrich) and helium at a flow rate of 1 mL/min as carrier gas. GC oven temperature started at 40 °C, after 10 min ramped at 3 °C/min to the final temperature of 200 °C. A blank followed by a standard mix was run after every ten samples. The data were recorded and analysed using Agilent MassHunter Qualitative Analysis. The volatile compounds were identified using the NIST 08 Mass Spectral Library and via comparison with the retention time and mass spectrum of their respective standards. A representation of a chromatogram from walnut kernel and oil volatile analysis with retention times is shown in Figure S1 and Table S1 (supplementary material). Results were expressed as µg of internal standard per kg of sample. Each sample of kernels and oil was analysed in triplicates.

### 2.14. Data Analysis

All data were tested by analysis of variance (ANOVA), and significant storage effects for each cultivar were followed by Tukey’s multiple comparison test at *p* ≤ 0.05. The heatmap of volatile compounds was generated using the normalized volatile concentration, obtained by the ratio of each volatile concentration and the sum of volatile compounds from the same sample. The significant chemical data were then related to one another using multivariate analysis. Principal component analysis (PCA) was applied to evaluate the chemical composition of both cultivars present in the study. Hierarchical cluster analysis (HCA) and partial least square regression discriminant analysis (PLS-DA) were applied to identify their characteristic properties, and to know which are associated with oxidative stability. Data analysis were performed by SPSS version 27.0 software (SPSS Inc., Chicago, IL, USA) and graphs were obtained using SigmaPlot version 12.5 (Systat Software Inc., San Jose, CA, USA).

## 3. Results and Discussion

### 3.1. Evolution of the Quality Parameters during Storage

Walnuts have one of the highest fat content among nuts and foods [11]. Fat content in kernels was significantly higher in ‘Chandler’ (65.5 g/100g dry weight) compared to ‘Howard’ (46.3 g/100g dry weight). Table 1 represents the changes in moisture content, FFA, PV, and UV absorbances at 232 nm (K_232_) and 268 nm (K_268_). Moisture is an essential factor in the quality of nuts, influencing their physical and chemical properties [21]. The moisture content in both ‘Chandler’ and ‘Howard’ kernels decreased significantly during storage (*p* < 0.000). Compared with ‘Chandler’, ‘Howard’ had a higher initial moisture content, experienced a sharp decrease within the first week, and had lower moisture content throughout the 28 weeks.

Hydrolysis of triacylglycerols (TAG) is due to the lipolysis (by lipases) or hydrothermal activity, resulting in the release of free fatty acids and hydrolytic rancidity [11]. Thus, FFA reflects the amount of fatty acids hydrolyzed from TAG. In this study, FFA was unchanged or maintained at 0.04 g 100g^−1^ of oleic acid for both cultivars during storage, likely due to the drying process and storage conditions that reduced moisture content and inactivated lipases and other enzymes activity [22,23].

PV measures the concentration of primary oxidation products (hydroperoxides) in fats and oils [11]. As oxidation progresses, PV peaks and then declines as the hydroperoxides breakdown into secondary oxidation products. In this study, PV increased significantly along with storage (*p* < 0.000) and was higher in the oil extracted from ‘Howard’ than it was from ‘Chandler’ (*p* < 0.000). In ‘Chandler’, PV ranged from 1.0 to 4.3 mEq O_2_kg^−1^ while in ‘Howard’, PV ranged from 1.8 to 6.4 mEq O_2_kg^−1^ between 0–28 weeks. PV of 3 mEq O_2_kg^−1^ is the maximum limit of acceptable quality of shelled walnuts [22]. ‘Howard’ reached 3.8 mEq O_2_kg^−1^ after 20 weeks of storage, ‘Chandler’ reached the same value after 24 weeks. According to the recognized PV limit of acceptable quality in kernels, ‘Chandler’ can be stored no longer than six months and ‘Howard’ no longer than four months under our studied storage condition.

K_232_ determines the peroxides present via the conjugated double bonds of the hydroperoxides. Both cultivars had similar ranges of K_232_, though values from ‘Chandler’ were consistently lower than those from ‘Howard’. K_232_ values of ‘Chandler’ ranged from 0.95 to 3.08 while ‘Howard’ ranged from 1.13 to 3.2. K_268_ measures the conjugated trienes from more advanced oxidation processes. Same as K_232_ values, K_268_ values were higher in ‘Howard’ than they were in ‘Chandler’ at all time points (*p* < 0.000). K_268_ values of ‘Chandler’ ranged from 0.07 to 0.23 while ‘Howard’ ranged from 0.13 to 0.29. As oxidation progresses during storage, increasing K_232_ and K_268_ trends were observed in both cultivars. 

### 3.2. Oil and Kernel Oxidative Stability during Storage

Oxidative stability gives an estimation of food susceptibility to oxidation; it can be used to provide information on the hypothetical stability of a product as it generally correlates with shelf life [24]. Walnut oil and kernels oxidative stability has been reported in previous studies, and different parameters of temperature and airflow have been applied [25,26,27,28,29]. We used the parameters advised by Metrohm Instruments specifically for walnut oil; the same method was applied to the oil and kernel to allow a comparison of both matrixes. Both oil and kernel oxidative stability decreased significantly for both cultivars during storage (Table 1). Oxidative stability of ‘Chandler’ oil ranged from 3.3 to 2.5 h while ‘Howard’ oil ranged from 3.3 to 2.2 h. The oxidative stability values obtained at the beginning of the study were within the same range described in literature when the same Rancimat parameters were applied to non-oxidized walnut oils [25,28].

Kernel oxidative stability even though presented much higher values; it followed the same trend as oil oxidative stability between cultivars and during storage. ‘Chandler’ demonstrated higher kernel oxidative stability than ‘Howard’ (*p* < 0.000). Kernel oxidative stability ranged from 13.4 to 6 h in ‘Chandler’ and from 11.7 to 2.5 h in ‘Howard’. Kernel oxidative stability significantly decreased when PV was close to the recognized parameters of acceptable quality of shelled walnuts of 3 O_2_kg^−1^ (2.6 O_2_ kg^−1^ in ‘Chandler’ at week 20 and 3.4 O_2_ kg^−1^ in ‘Howard’ at week 16). Differences between cultivars in PV, K_268_, and kernel oxidative stability were significant before storage, showing that these methods can be used to predict samples susceptibility to oxidation and to evaluate storage time and conditions. As we recently demonstrated [29], direct measurements of oxidative stability using walnut kernels can open the possibility of a shelf-life assessment considering all nut matrix.

### 3.3. Effect of Cultivar and Storage on Kernel Surface Darkness

Kernel surface darkness (Table 1) was included in the measured variables not only because it is a quality attribute perceived by consumers, but also its direct relation to the their chemical composition (e.g., phenolics) or moisture content [30]. Since phenols are mainly found in nut pellicles [31], their enzymatic or chemical oxidation, proven to be cultivar dependent, can cause browning of the kernels during storage [32]. All kernels from both cultivars were classified as ‘extra light’ (1 ± 0) for the first 2 weeks of storage. No significant differences were found between ‘Chandler’ and ‘Howard’ for the first month of storage, yet in the following weeks ‘Howard’ demonstrated significant darker kernels compared to ‘Chandler’. After 28 weeks of storage, ‘Chandler’ presented 2.8 of kernel darkness, meaning that some kernels were still ‘light’ or ‘extra light’. ‘Howard’ presented an average of 3.5 and all kernels were classified as ‘amber’ or ‘dark amber’. Although no differences were found between cultivars before and during the first 4 weeks of storage, our results are consistent with previous reports where ‘Chandler’ was described as the brightest cultivar derived from the Californian breeding program [33] and ‘Howard’ demonstrated a higher predisposition to dark kernels during storage when compared to ‘Chandler’ [29,34]. 

### 3.4. Effect of Cultivar and Storage on Walnut Oil Fatty Acid Profile

The fatty acid composition is often used as a potential predictor for stability, physical properties, and nutritional value in food. Fatty acids double bonds can be easily attacked by free radicals [5] with autoxidation rates of 1:40:100 for oleic, linoleic, and linolenic methyl esters, respectively [35]. Walnuts contain the higher concentrations of polyunsaturated fatty acids, linoleic (C18:2), and linolenic (C18:3) acids, than other nuts in the market, such as almonds or pistachios [4]. Table 2 contains the saturated fatty acid (SFA), monounsaturated fatty acids (MUFA), polyunsaturated fatty acids (PUFA), and linoleic/linolenic acid ratio (O6/O3) for ‘Chandler’ and ‘Howard’ during storage. A significant difference between the two cultivars was observed for MUFA and linoleic/linolenic ratio. On average, ‘Chandler’ had a higher MUFA and MUFA/PUFA ratio; and lower linoleic/linolenic ratio compared to ‘Howard’. Even though ’Howard’ was described in previous research as the highest polyunsaturated fatty acid among cultivars grown in the USA [36], the difference between cultivars was not significant in this study. Linoleic acid was the fatty acid found in the highest concentration (58–59%), followed by oleic (14–15%) and linolenic acids (14–15%). A previous study with ‘Chandler’ kernels stored at low temperatures (1 °C and 20 °C) demonstrated a decrease of unsaturated fatty acids while saturated fatty acids remain stable during 12 months of storage [37]. In this study, the changes in individual fatty acids did not follow a clear pattern during storage (Table S1, Supplementary Material). While rancidity may be perceived after months of storage and affects the quality negatively, the potential health benefit attributed to their unaffected fatty acid profile may still be intact. 

### 3.5. Changes in Tocopherols Concentration during Storage

Tocopherols are natural antioxidants that inhibit lipid oxidation in vegetable oils [38], and walnut oil is known for its high tocopherols content [39]. Antioxidation reactions lead to tocopherols degradation and consumption, decreasing their concentration in food over time [40,41]. The individual tocopherol concentration and the sum of tocopherols are shown in Table 2. The main tocopherol detected on the samples was γ-tocopherol, representing 84.7% and 81.2% of the total tocopherols in ‘Chandler’ and ‘Howard’, respectively. In our study, during 28 weeks of storage, total tocopherol concentrations in ‘Chandler’ changed −1.26-fold from 494 mg kg^−1^ to 391 mg kg^−1^. Similarly, total tocopherol concentrations in Howard’ changed −1.34-fold from 386 mg kg^−1^ to 289 mg kg^−1^. A study of ten walnut cultivars in Turkey reported similar ranges of tocopherols for Chandler (313.59 mg kg^−1^) and Howard (213.29 mg kg^−1^) [39].

### 3.6. Changes in Kernel Phenols during Storage

Total phenolic content of walnuts can be a useful criterion for overall walnut quality evaluation due to their contribution to the color, taste, flavor characteristics, and overall health benefits [4]. Total phenols in ‘Chandler’ and ‘Howard’ kernels during storage are represented in Table 2. ‘Chandler’ had significantly higher total phenolic concentration than ‘Howard’ did, in both initial kernels and kernels after 28 weeks of storage. Both cultivars experienced a significant loss in phenols during storage, as most of these may be lost to fend off oxidation. ‘Chandler’ kernels started at 12,402 µg g^−1^ and decreased to 10,466 µg g^−1^, while ‘Howard’ started at 11,511 µg g^−1^ and decreased to 8325 µg g^−1^. The total phenolic concentration found in the present study is within the same range as those previously reported for ‘Chandler’ and ‘Howard’ grown in Spain, Greece, and Turkey and for ‘Chandler’ stored at 20 °C for 12 months [19,30,37,39]. The rate of phenol decreases during storage was higher in ‘Howard’ (−1.4-fold) than it was in ‘Chandler’ (−1.2-fold). 

### 3.7. Changes in Oil Phenols during Storage

Contrary from the phenols in kernels, ‘Howard’ oil had a higher initial phenolic concentration than the oil from ‘Chandler’. During storage, as expected, phenols are used up to fend off oxidation, ‘Chandler’ decrease oil phenol concentration by −1.5-fold, similarly to kernels. However, ‘Howard’ demonstrated higher losses with −10.2-fold oil phenol concentration after 7 months of storage. Phenol decreased more rapidly in oil than in kernel, and in ‘Howard’ than in ‘Chandler’. Walnut oil presented much lower phenol concentration compared to kernels, due to their low oil solubility [5]. Therefore, oxidation protection of the phenolics is much more limited in oil than they are in the kernels. 

### 3.8. Changes in Kernel Volatile Concentration during Storage

**Figure 1:**
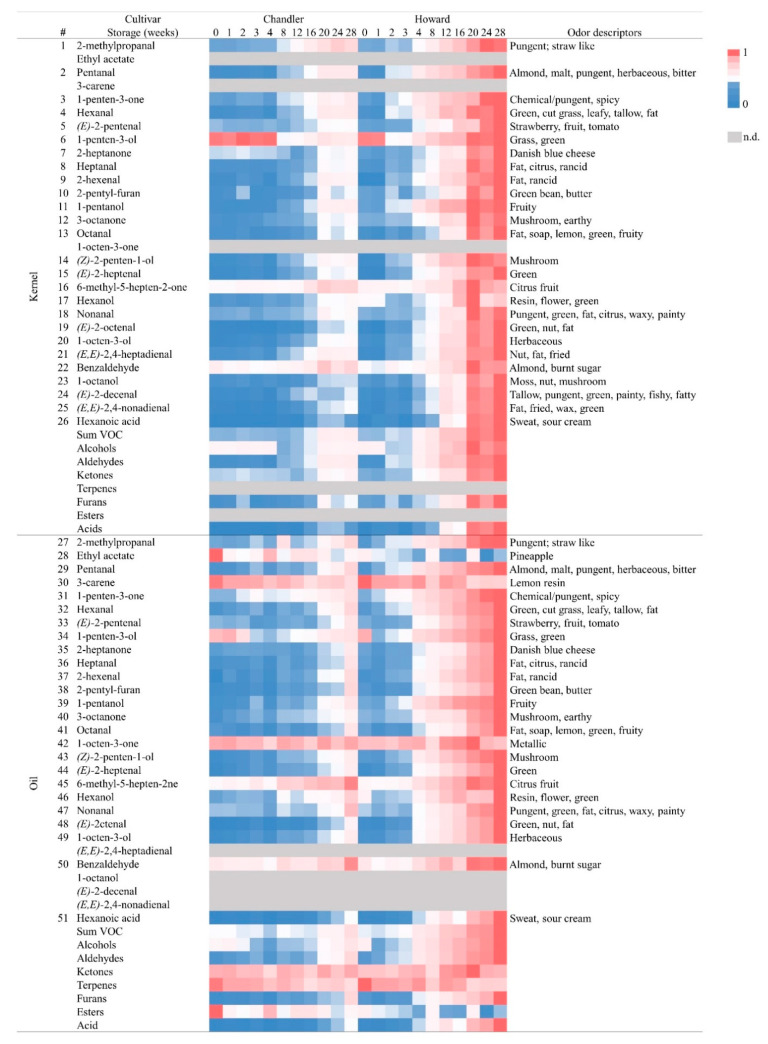
Heatmap showing changes in the individual volatile compound concentration (*n* = 3) at 11-time points during 28 weeks of storage of Chandler and Howard kernels. Concentrations of each volatile are relative to the maximum concentration of the same volatile detected across samples. Blue color represents the lower concentration (0), while dark pink color represents the higher volatile concentration (1). Compounds non detected (n.d.) are represented in grey color. Odor descriptors from database flavornet.org and other authors [45,46,56].

When lipid matrix of walnuts is exposed to heat and light, the hydrogen atom of double bond is extracted, and alkyl radicals are formed. These free radicals react with oxygen; peroxy radicals are formed by removing hydrogen atoms from another unsaturated fatty acids, leading to the formation of primary oxidation products called hydroperoxides by the mechanisms of initiation, propagation, and termination [42]. These primary oxidation products further break down into carbonyl compounds such as aldehyde, ketones, and alcohols [7]. Although hydroperoxides are generally tasteless and odorless, their oxidation products can have an impact on flavor. Some volatile oxidation products can alter the flavor of vegetable oils at concentrations lower than 1 ppm [7]. Figure 1 shows a heatmap describing the changes in kernel volatile compound concentration for 28 weeks. Oxidative degradation of oleic acid yields nonanal and octanal; linoleic acid can be easily oxidized to produce hexanal, pentanal, heptanal, and *(E)*-2-hexenal; and linolenic acid is a precursor of *(Z,Z)*-2,4-heptadienal, *(E)*-2-heptenal, and 2-methylpropanal. The oxidative degradation rate of linolenic acid is much faster than that of linoleic acid and oleic acid [43]. Due to the low odor threshold of the volatiles formed during oxidation, differences between volatile profile can be expected in walnut samples, while little or no differences were founded in the fatty acid profile during storage [35]. At the same time, tocopherols, known to inhibit lipid oxidation [38], significantly decrease during storage and are significantly different between cultivars. The total concentration of volatiles in kernels range from 3.4 to 19.6 mg kg^−1^ in ‘Chandler’ and from 3.8 to 105.72 mg kg^−1^ in ‘Howard’. As presented in Figure 1, volatile concentrations started to increase after eight to twelve weeks of storage in ‘Chandler’, while the increase occurred after two to three weeks in ‘Howard’. From the 26 volatiles identified through analytical standards, hexanal and 1-penten-3-ol demonstrated the highest concentration in kernels from ‘Chandler’ and ‘Howard’ cultivars (Tables S2 and S3 on Supplementary Material). However, the cultivars presented a different hierarchy of volatiles at the beginning of the study, demonstrating the genetic influence. In particular, ‘Chandler’ presented 1-penten-3-ol > hexanal > 2-heptanone > *(E)*-2-pentenal > 2-pentyl-furan as the volatiles with highest concentrations, while ‘Howard’ presented 1-penten-3-ol > 1-hexanol > hexanal > 2-pentyl-furan > 1-pentanol with the highest concentrations. The hierarchy of the volatiles with highest concentration also changed during storage. Specifically, ‘Chandler’ presented hexanal > 1-pentanol > pentanal > 1-octen-3-ol > 2-pentyl-furan with the highest concentrations after 28 weeks, while ‘Howard’ presented hexanal > 2-pentyl-furan > 1-pentanol > 1-octen-3-ol > *(E)*-2-octenal as the volatiles with highest concentrations after the same period. Contrary to previous studies on walnut cultivars from Ukraine, China, and Chile [44], 1-octen-3-one, known for its metallic and mushroom odor descriptors [45,46] was not detected on our samples.

The detection of rancidity off-flavors depends on the type and degree of oxidation. In previous studies, hexanal was monitored to assess the oxidation degree in nut samples [9,47,48]. In roasted almonds and olive oil samples, the quantification of hexanal and nonanal were shown to be enough to detect oxidation before a sensory panel gives the rancid attribute [43,48]. The low threshold value of the volatile aldehydes produced from oxidized linolenate, such hexanal, make these compounds more relevant at initial stages of oxidation [43,49,50,51]. In this study, among stored samples, hexanal was the volatile present at the highest concentration, while 1-octen-3-ol demonstrated the biggest increase in the kernels of both cultivars during storage. Along with storage, 1-octen-3-ol, pentanal, hexanal, 2-pentyl-furan, and 1-penten-3-ol increased 72, 44, 43, 14, and 0.2-fold, respectively, in Chandler kernels, and 185, 146, 145, 57, 1.1-fold, respectively, in Howard kernels. Previous studies on walnut oil accelerated oxidation have shown hexanal with the highest concentration even at the starting point of oxidation [52], others have also shown that hexanal is an important contributor for the distinctive walnut kernel aroma [53]. Lipoxygenases (LOXs) catalyze the hydroperoxidation of polyunsaturated fatty acids, such as linoleic and linolenic acids, which are abundantly accumulated in walnut kernels, with consequent production of 9- or 13- hydroperoxides. 13-LOXs give origin to hexanal and *(E)*-2-hexenal, two volatiles included in this study. Moreover, walnuts are known for their prevalence of 13-LOXs compared to those observed in other nuts, such as almonds [31,54]. Therefore, the baseline concentration of hexanal in kernel samples may be generated through the lipoxygenase pathway during kernel processing (e.g., drying). Since the studied kernels underwent the same processing and storage condition for both cultivars, it suggests that hexanal concentration at the beginning of the study and during storage may be variety dependent.

### 3.9. Changes in Oil Volatile Concentration during Storage

Contrary to the kernels, only 3-carene (terpene), ethyl acetate (ester), and 1-octen-3-one (ketone) were identified in the oil extracted from kernels after storage. *(E,E)*-2,4-Heptadienal, 1-octanol, *(E)*-2-decenal, and *(E,E)*-2,4-nonadienal were not detected in the oil (Tables S4 and S5 on Supplementary Material). These volatiles have odor descriptors of ‘nutty’, ‘tallow’, ‘fried’, and ‘fat’, and their absence in the oil may indicate that walnut oil does not have the same distinctive odor as the kernels. For the volatiles identified in both kernels and oils, concentrations were similar, or lower in the oil compared to the kernel. This was expected, since the vigorous mixing of oil with water during pressing extraction may gradual decrease the water-soluble oxygenated compounds and increase terpenes in oils [55]. Among the volatile compounds detected, 1-octen-3-one was presented at highest concentration, followed by 1-penten-3-ol on both cultivars. Even though 1-octen-3-one did not change significantly during storage, it is present above its odor threshold of 10 µg/kg oil, which may also contribute to a distinctive oil flavor compared to kernel flavor [45]. At harvest, walnut oil volatile concentrations were higher in ‘Howard’ compared to ‘Chandler’, except for ethyl acetate that was present in higher concentration in ‘Chandler’. The change during storage of the relative volatile concentration of each sample is represented in the heatmap, Figure 1. As previously described for kernels, volatile compounds concentration in the oil increased during storage, except for 3-carene, a terpene responsible for lemon odor. Volatiles increased relatively to the maximum described in each sample and followed the same trend as kernel volatiles for both cultivars studied. Oil volatiles doubled their concentration after 8 to 12 weeks of storage for ‘Chandler’, and before one month of storage for ‘Howard’. It is important to note that volatiles concentration during storage increased more in ‘Howard’ (represented in dark pink color in the heatmap, Figure 1) than in ‘Chandler’, for both kernels and oil. 

### 3.10. PCA Analysis

The results shown previously indicate that the chemical composition of walnut kernel and oil were affected by the storage. Moreover, oxidative stability, PV, K_232_, and K_236_ are higher in ‘Howard’ than in ‘Chandler’. Using PCA and HCA, we aimed to distinguish different oxidation levels and understand which chemical analysis and compounds are better related to kernel oxidative stability. These compounds might be better indicators of kernel oxidative stability during storage.

**Figure 2:**
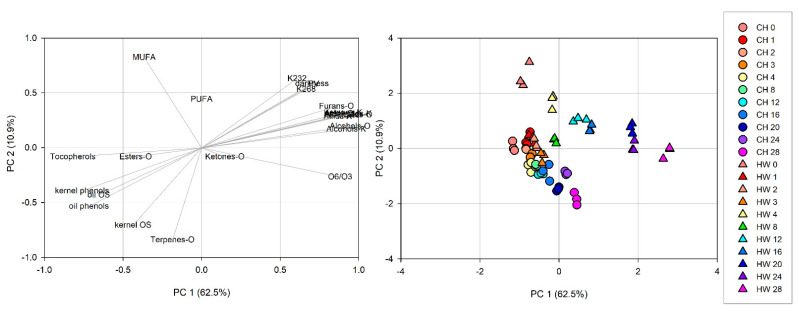
Principal component analysis of data ‘Chandler’ (CH) and ‘Howard’ (HW) kernel and oil during 28 weeks of storage. (**A**). Loading plot for PC1 and PC2 contributing mass peaks and their assignments labelled. (**B**). Score plot of PC1 versus PC2 scores of the samples.

Chemical composition was analyzed using principal component analysis (PCA) to explore the relative variability within the different walnut samples. PCA was generated with a correlation matrix of 66 observation from both walnut cultivars against 24 independent variables (based on the quality parameters (PV, K_232_, K_268_, kernel OS, oil OS), pellicle darkness, kernel and oil phenols, fatty acid profile (MUFA, PUFA, O_6_/O_3_), and volatiles classes from kernel and oil (Table 1 and Table 2, and Figure 1). As shown in Figure 2, two principal components were generated in the PCA with an eigenvalue greater than 1, account for 73.4% (62.5% for PC 1 and 10.9% for PC2) of the total variance. Kaiser-Meyer-Olkin measure was 0.886, and significance (*p* < 0.001) given by Bartlett’s test of sphericity. Ketones, esters, and PUFA present in the oil show values lower than 0.3 in the correlation matrix with all other values. This may be an indication than these variables can be further reduced in the analysis, since they are not correlated with other measurements included in this manuscript to describe walnut oxidation. The loading plot of PCA (Figure 2A) using varimax rotation method with Kaiser normalization allows for an easy visualization of the variable distribution and a better interpretation of the loading factors. Variables that define lipid oxidation such PV, K_232_, and K_268_ are together with pellicle darkness and volatile compounds from kernel and oil positioned most positively along PC1, whereas tocopherols and phenols, known for their antioxidant capacity contributed mostly negatively along PC2. Subsequently, analyzing the loading plots we could identify the first factor PC1 as the ‘high oxidation markers’, while the second factor PC2 as the ‘low oxidation markers’ in walnuts. The score plot of the samples (Figure 2B) represents the samples distributed according with the loading plot; symbols distinguish cultivar while color represent the storage time groups. ‘Chandler’ samples are more closely together towards PC2 (‘low oxidation markers’) than ‘Howard’, which are consistent with more tocopherols, phenols, and OS. After storage, ‘Chandler’ samples are distributed closer together than ‘Howard’ samples, indicating less chemical composition change in ‘Chandler’ during storage compared to ‘Howard’. 

### 3.11. Hierarchical Cluster Analysis (HCA)

**Figure 3:**
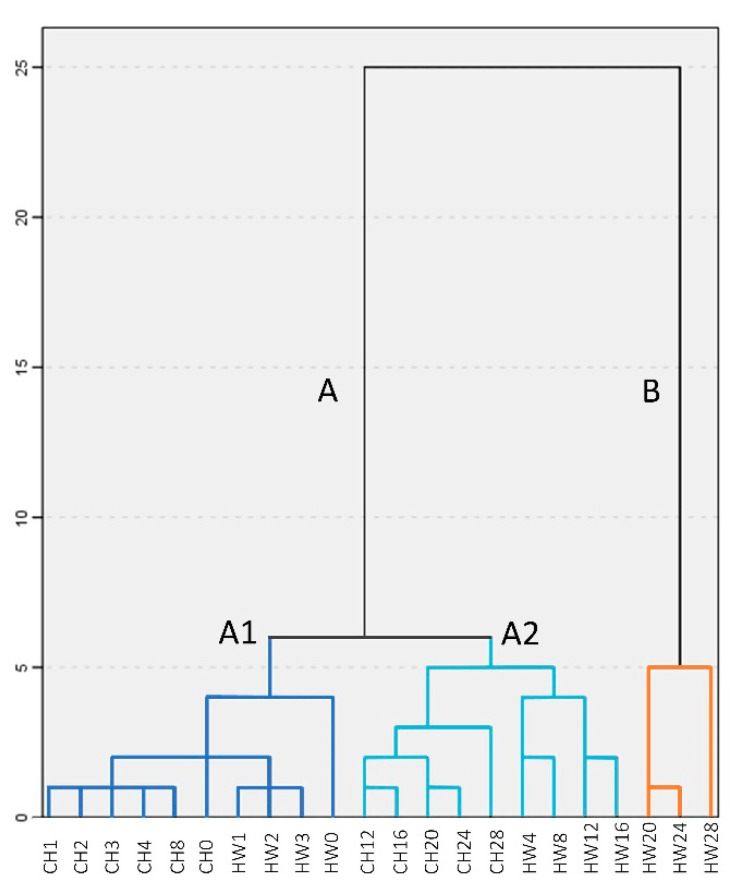
Hierarchical dendrogram for ‘Chandler’ (CH) and ‘Howard’ (HW) samples from 28 weeks of storage.

To further explore the possibility to separate samples into distinctive oxidation groups using the variables measured, HCA was performed. The two factors extracted from PCA were applied to HCA to explore the heterogeneity of the samples. As illustrated in Figure 3, the samples can be divided into two clear clusters: Groups A (lowest oxidation) and B (highest oxidation). Two sub-clusters were detected under group A: A1 (no oxidation) and A2 (low oxidation). The grouping results are similar with the results of PCA (Figure 2B), which illustrates that samples with longer storage time differ from those of short storage time, especially in ‘Howard’ samples. Though the results from PCA and HCA are useful to preliminarily distinguish kernel oxidation levels, sample size is limited and does not allow further application (e.g., different cultivars).

### 3.12. Partial Least Squares-Discriminant Analysis (PLS-DA) for Volatile Compounds Discrimination

**Figure 4:**
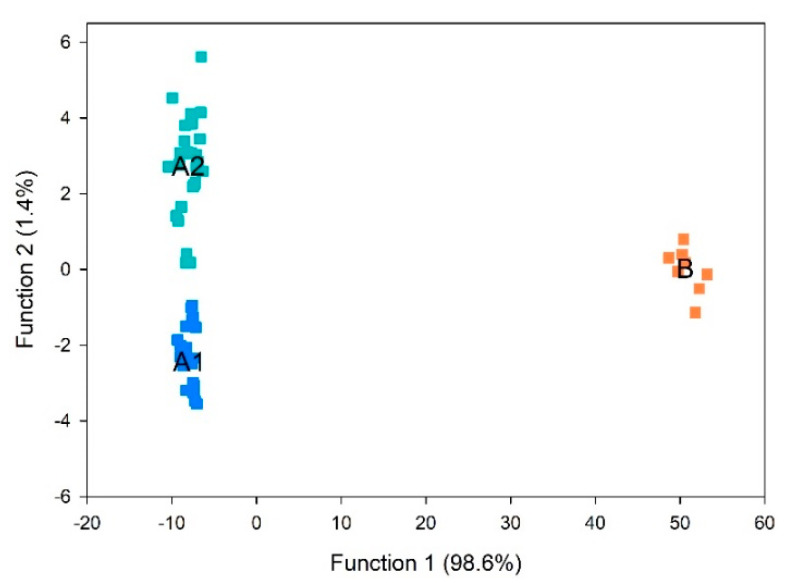
Canonical discriminant factors from the groups A1 (no oxidation), A2 (lowest oxidation), and B (highest oxidation).

As demonstrated in the loading plot from PCA (Figure 2A), increases in volatile compounds in walnuts were amongst the oxidation markers. This supports our hypothesis that walnut oxidation can be tracked by measuring kernel volatile compounds, reducing sample amount, time, and effort for oil extraction necessary for commonly used methods, such FFA and PV. To further explore this hypothesis and to compare volatile profiles among the sample groups, PLS-DA was performed. PLS-DA is a supervised multivariate analysis tool that can separate the predictive variation and characteristic markers for each cluster (Gao et al. 2018), which was applied to identify the characteristic volatile for each cluster from Figure 3. Discrimination between walnut samples was statistical significance (*p* < 0.001). The results indicated that two discriminant functions were optimal to build the model for discriminating samples. The scores and correlation loadings obtained for the first two latent components of the calculated PLS-DA model were combined in a plot (Figure 4). The first two components of PLS-DA accounted for 100% of the total variance among samples, with most of the variance explained by the first function. PLS-DA properly separated group A1 (no oxidation) from group A2 (lowest oxidation), and group B (highest oxidation) was distinguished from the other two groups. These results indicated that the groups previously defined in HCA could be discriminated using kernel volatile compounds. 

To identify the specific volatiles that most contribute for the group separation, a stepwise method was applied. In twenty-one steps, thirteen volatile compounds that contribute for discrimination between oxidation groups were established (Table 3). We found that 2-Methylpropanal, pentanal, hexanal, (E)-2-pentenal, 3-octanone, octanal, (Z)-2-penten-1-ol, hexanol, (E)-2-octenal, 1-octen-3-ol, benzaldehyde, (E,E)-2,4-nonadienal, and hexanoic acid in kernels can be used to distinguish different oxidation levels and as markers for oxidation in walnuts. The within-sample robustness of the predictive properties of the derived model was assessed using leave-one-out cross-validation. Under leave-one-out cross-validation, 64/66 (97%) grouped cases were correctly classified (Table 4), with two samples from group A2 being mis-classified as group A1.

## 4. Conclusions

Walnuts are susceptible to oxidative rancidity due to high fat content and proportion of unsaturated fatty acids. Under the same storage condition, oil extracted from ‘Howard’ kernels had higher values of PV, UV absorbances, as well as lower phenols, tocopherols, and MUFA, comparing to oil extracted from ‘Chandler’. Higher concentrations of phenols and tocopherols in ‘Chandler’ likely contribute to their stability comparing to ‘Howard’.

PCA, HCA, and PLS-DA were successfully employed to assess and compare the utility of different oxidation methods, including volatiles in kernels and oil. Kernel volatile compounds can distinguish samples with different storage time for ‘Chandler’ and ‘Howard’. This work shows that volatile measurements of the kernels can be applied to help growers and processors to assess walnuts level of oxidation at harvest and during storage without oil extraction. Future work needs to investigate the potential utility of these parameters as oxidation markers in walnut kernels under different and practical storage scenarios as well as their acceptable limits and relation to their sensory attributes.

## Figures and Tables

**Table 1:** Quality parameters in Chandler and Howard cultivars during 28 weeks of storage.

Cultivar	Storage (Weeks)	Moisture Content	Oil FFA	Oil PV	Oil K_232_	Oil K_268_	Kernel OS	Oil OS	Kernel Darkness
Chandler	0	2.5 ± 0.25 a	0.03 ± 0.01	0.9 ± 0.1 d	0.95 ± 0.12 h	0.07 ± 0.01 e	13.3 ± 0.03 a	3.3 ± 0.04 a	1.0 ± 0.00 g
	1	2.4 ± 0.22 ab	0.03 ± 0.00	1.2 ± 0.01 d	1.14 ± 0.01 g	0.09 ± 0.00 de	13.4 ± 0.45 a	2.7 ± 0.01 cd	1.0 ± 0.00 g
	2	2.2 ± 0.23 abc	0.03 ± 0.00	1.3 ± 0.10 d	1.33 ± 0.04f g	0.12 ± 0.02 bc	12.8 ± 0.12 a	3.0 ± 0.14 b	1.0 ± 0.00 g
	3	2.3 ± 0.22 abc	0.04 ± 0.00	1.3 ± 0.11 d	1.43 ± 0.01 ef	0.11 ± 0.01 bcd	12.4 ± 0.64 ab	2.8 ± 0.12 bc	1.1 ± 0.23 g
	4	2.2 ± 0.32 abc	0.04 ± 0.01	1.9 ± 0.23 c	1.40 ± 0.12 f	0.10 ± 0.02 cd	12.5 ± 0.47 ab	2.9 ± 0.03 b	1.3 ± 0.46 fg
	8	1.8 ± 0.05 c	0.04 ± 0.00	2.3 ± 0.36 bc	1.60 ± 0.04 e	0.11 ± 0.01 cd	12.6 ± 0.13 a	2.8 ± 0.01 bcd	1.6 ± 0.51 ef
	12	1.8 ± 0.03 bc	0.03 ± 0.01	2.6 ± 0.14 b	2.00 ± 0.21 d	0.12 ± 0.03 c	13.2 ± 0.19 a	2.7 ± 0.04 bcd	1.8 ± 0.44 de
	16	1.8 ± 0.13 c	0.03 ± 0.00	2.7 ± 0.48 b	2.40 ± 0.13 c	0.14 ± 0.01 b	12.7 ± 1.70 a	2.7 ± 0.21 bcd	2.0 ± 0.23 cd
	20	1.7 ± 0.15 c	0.03 ± 0.00	2.6 ± 0.19 b	2.85 ± 0.03 b	0.21 ± 0.01 a	10.9 ± 2.49 bc	2.6 ± 0.08 cde	2.1 ± 0.35 bc
	24	1.1 ± 0.16 d	0.04 ± 0.00	3.8 ± 0.20 a	3.01 ± 0.07 ab	0.21 ± 0.01 a	10.6 ± 0.27 c	2.4 ± 0.09 e	2.4 ± 0.49 b
	28	1.0 ± 0.03 d	0.04 ± 0.00	4.4 ± 0.61 a	3.08 ± 0.06 a	0.23 ± 0.01 a	6.00 ± 0.40 d	2.5 ± 0.10 de	2.8 ± 0.47 a
Howard	0	3.0 ± 0.06 a	0.03 ± 0.00	1.6 ± 0.32 g	1.13 ± 0.04 f	0.13 ± 0.04 e	10.9 ± 0.91 a	3.3 ± 0.56 a	1.0 ± 0.00 g
	1	1.3 ± 0.43 b	0.03 ± 0.00	1.7 ± 0.17 fg	1.38 ± 0.03 e	0.17 ± 0.01 c	10.9 ± 0.08 a	3.0 ± 0.49 ab	1.0 ± 0.00 g
	2	1.4 ± 0.18 b	0.03 ± 0.00	1.7 ± 0.28 fg	1.66 ± 0.03 d	0.17 ± 0.02 cde	11.1 ± 0.67 a	2.8 ± 0.04 abc	1.0 ± 0.00 g
	3	1.3 ± 0.03 b	0.03 ± 0.01	2.1 ± 0.21 ef	1.59 ± 0.11 d	0.12 ± 0.04 de	11.0 ± 0.34 a	2.5 ± 0.12 bcd	1.1 ± 0.27 g
	4	1.2 ± 0.07 b	0.03 ± 0.00	2.3 ± 0.11 de	1.64 ± 0.04 d	0.15 ± 0.03 cde	11.7 ± 1.09 a	2.5 ± 0.12 bcd	1.7 ± 0.48 f
	8	1.2 ± 0.03 b	0.04 ± 0.00	2.6 ± 0.46 de	1.84 ± 0.15 c	0.16 ± 0.03 cde	11.7 ± 0.82 a	2.4 ± 0.11 bcd	2.0 ± 0.16 e
	12	0.9 ± 0.07 bc	0.04 ± 0.00	2.8 ± 0.36 d	2.66 ± 0.14 b	0.18 ± 0.01 c	10.4 ± 0.58 a	2.4 ± 0.04 bcd	2.2 ± 0.37 de
	16	1.1 ± 0.12 bc	0.04 ± 0.01	3.4 ± 0.32 c	2.56 ± 0.03 b	0.16 ± 0.02 c	8.20 ± 1.86 b	2.4 ± 0.08 bcd	2.4 ± 0.48 cd
	20	0.9 ± 0.03 bc	0.03 ± 0.00	3.8 ± 0.18 bc	3.11 ± 0.06 a	0.24 ± 0.01 b	7.60 ± 1.05 b	2.0 ± 0.10 cd	2.6 ± 0.51 c
	24	0.5 ± 0.06 c	0.04 ± 0.01	4.0 ± 0.01 b	3.04 ± 0.11 a	0.25 ± 0.01 b	4.80 ± 1.31 c	2.2 ± 0.16 cd	2.9 ± 0.37 b
	28	0.5 ± 0.17 c	0.04 ± 0.01	6.4 ± 0.20 a	3.21 ± 0.06 a	0.29 ± 0.05 a	2.50 ± 0.24 d	2.2 ± 0.01 d	3.5 ± 0.51 a
Chandler		1.9 ± 0.50 a	0.03 ± 0.01	2.2 ± 1.10b	1.85 ± 0.13 b	0.07 ± 0.70 b	11.9 ± 2.23 a	2.8 ± 0.25 a	1.8 ± 0.67 a
Howard		1.3 ± 0.72 b	0.03 ± 0.01	2.9 ± 1.35 a	2.11 ± 0.18 a	0.09 ± 0.73 a	9.11 ± 3.07 b	2.5 ± 0.41 b	2.3 ± 0.79 b

Moisture content expressed as g kg^−1^; free fatty acid (FFA) as g 100 g^−1^ of oleic acid; peroxide value (PV) as meq O_2_ kg^−1^; coefficient of specific extinction at 232nm (K_232_); coefficient of specific extinction at 268nm (K_268_); kernel and oil oxidative stability (OS) expressed in hours; kernel color expressed in DFA scores. Letters denote significant differences between storage weeks within the same cultivar samples and significant differences between cultivars in the last two rows (*ρ* < 0.001).

**Table 2:** Total phenols, tocopherols, and fatty acids in Chandler and Howard cultivars during 28 weeks of storage.

Cultivar	Storage (Weeks)	Kernel Phenols	Oil Phenols	δ-t	γ-t	α-t	∑Tocopherols	SFA	MUFA	PUFA	O6/O3
Chandler	0	12402 ± 502.8 a	102 ± 1.1 a	59 ± 0.2 a	411 ± 12.3 a	24 ± 0.5 a	494 ± 11.6 a	7.4 ± 1.1	20.7 ± 0.3	72 ± 0.8	3.91 ± 0.00
	1	12081 ± 511.0 a	100 ± 3.2 a	58 ± 0.1 a	387 ± 17.3 a	19 ± 0.6 b	464 ± 18 a	7.4 ± 0.3	19.9 ± 0.5	72.7 ± 0.1	3.99 ± 0.01
	2	12316 ± 569.4 a	105 ± 1.4 a	58 ± 0.0 a	387 ± 1.20 abc	17 ± 1.3 b	462 ± 2.5 ab	6.7 ± 0.1	20.6 ± 0.1	72.7 ± 0.0	3.97 ± 0.00
	3	11929 ± 172.4 ab	98 ± 3.0 a	59 ± 3.6 ab	379 ± 10.7 abcd	10 ± 1.1 c	447 ± 13.2 abc	6.3 ± 0.3	20.7 ± 0.1	73.1 ± 0.2	4.02 ± 0.00
	4	10973 ± 369.0 bc	90 ± 1.0 b	63 ± 2.4 ab	400 ± 15.5 ab	9 ± 0.3 c	472 ± 18.2 ab	7.1 ± 0.9	21.1 ± 0.3	71.8 ± 0.6	3.99 ± 0.00
	8	10986 ± 147.1 bc	83 ± 1.9 c	63 ± 0.6 ab	383 ± 10.7 ab	7 ± 0.2 d	453 ± 11.1 ab	6.0 ± 0.6	20.9 ± 0.1	73.2 ± 0.5	3.97 ± 0.01
	12	10870 ± 162.7 bc	84 ± 2.5 bc	59 ± 0.6 ab	348 ± 5.7 bcde	4 ± 0.5 e	411 ± 6.8 bcd	5.9 ± 0.4	20.9 ± 0.0	73.2 ± 0.4	3.97 ± 0.01
	16	10564 ± 337.0 c	87 ± 2.1 bc	56 ± 1.3 ab	336 ± 8.1 cde	0 ± 0.0 f	392 ± 9.3 cd	7.9 ± 1.6	20.4 ± 0.4	71.6 ± 1.2	3.98 ± 0.00
	20	10006 ± 546.4 c	83 ± 2.5 c	58 ± 5.5 ab	334 ± 10.7 de	0 ± 0.0 f	392 ± 16.2 cd	6.4 ± 0.7	21.2 ± 0.1	72.4 ± 0.6	3.91 ± 0.01
	24	10218 ± 238.2 c	69 ± 3.0 d	52 ± 5.1 b	328 ± 20.1 e	0 ± 0.0 f	380 ± 25.2 d	7.0 ± 0.2	20.5 ± 0.1	72.4 ± 0.2	4.03 ± 0.01
	28	10466 ± 146.6 c	67 ± 0.9 d	53 ± 0.5 ab	337 ± 11.2 cde	0 ± 0.0 f	391 ± 11.7 d	6.3 ± 0.1	21 ± 0.1	72.7 ± 0.1	3.95 ± 0.01
Howard	0	11511 ± 1101.4 a	143 ± 1.9 a	56 ± 2.1 a	309 ± 5.5 a	21 ± 0.6 a	386 ± 8.20 a	10 ± 0.9	18.5 ± 0.9	71.2 ± 0	4.21 ± 0.11
	1	10880 ± 321.6 ab	142 ± 1.2 a	51 ± 1.2 a	308 ± 20 a	15 ± 1.9 b	375 ± 23.2 a	6.1 ± 0.1	20.3 ± 0.0	73.6 ± 0.1	4.04 ± 0.00
	2	10830 ± 264.7 ab	123 ± 1.1 b	56 ± 2.1 ab	318 ± 11.5 ab	12 ± 1.1 c	385 ± 12.6 a	6.2 ± 0.2	20.4 ± 0.1	73.5 ± 0.2	4.03 ± 0.01
	3	10694 ± 523.6 abc	92 ± 6.2 c	51 ± 0.9 ab	281 ± 5.4 abc	11 ± 1.1 cd	343 ± 7.4 ab	4.3 ± 0.8	20.6 ± 0.2	75.1 ± 0.6	4.01 ± 0.02
	4	10150 ± 483.4 abcd	88 ± 6.0 cd	47 ± 0.9 abc	265 ± 8.3 cd	8 ± 1.1 d	321 ± 8.5 bc	11 ± 0.2	19.3 ± 0.2	70.1 ± 0.0	4.09 ± 0.07
	8	10592 ± 319.5 abc	81 ± 1.3 d	49 ± 2.0 abc	268 ± 6.3 bcd	n.d.	317 ± 8.2 bc	7.5 ± 0.1	20.2 ± 0.1	72.3 ± 0.1	4.03 ± 0.01
	12	10237 ± 693.3 abcd	44 ± 2.3 e	43 ± 5.8 bc	232 ± 11.9 d	n.d.	275 ± 17.7 c	8.4 ± 0.2	19.8 ± 0.0	71.8 ± 0.2	4.07 ± 0.01
	16	9699 ± 166.0 bcde	40 ± 2.6 e	39 ± 1.9 c	238 ± 13.5 d	n.d.	277 ± 15.4 c	6.2 ± 0.2	20.3 ± 0.1	73.5 ± 0.3	4.24 ± 0.00
	20	9280 ± 492.8 cde	39 ± 1.2 e	42 ± 4.6 bc	254 ± 6.0 cd	n.d.	296 ± 10.6 bc	7.1 ± 0.2	20.3 ± 0.0	72.7 ± 0.2	4.22 ± 0.00
	24	9055 ± 268.6 de	25 ± 1.3 f	38 ± 2.9 c	246 ± 7.0 cd	n.d.	284 ± 10.0 c	6.2 ± 0.2	20.6 ± 0.0	73.2 ± 0.1	4.21 ± 0.01
	28	8325 ± 258.4 e	14 ± 3.2 g	40 ± 1.6 bc	248 ± 6.3 cd	n.d.	289 ± 7.9 c	5.4 ± 0.7	20.6 ± 0.3	74.0 ± 0.3	4.21 ± 0.00
Chandler		11165 ± 895.4 a	88 ± 12.4	58 ± 3.9 a	366 ± 30.6 a	8 ± 8.3	432 ± 40.5 a	6.7 ± 0.8	20.7 ± 0.4 a	72.5 ± 0.7	3.97 ± 0.04 b
Howard		10114 ± 1003.7 b	76 ± 45.2	47 ± 6.6 b	270 ± 30.5 b	6 ± 7.5	322 ± 43.3 b	7.1 ± 1.9	20.1 ± 0.7 b	72.8 ± 1.4	4.12 ± 0.10 a

Total phenols in kernel expressed in µg g^−1^; the sum (ΣTocopherols) and individual tocopherol: Delta (δ-t), gamma (γ-t), and alpha (α-t) content and selected fatty acid (% of total fatty acid detected) in oil from extracted at eleven-time points for 28 weeks storage time. Tocopherol concentrations are expressed in mg kg^−1^ of oil. SFA, saturated fatty acid; UFA, unsaturated fatty acid; MUFA, monounsaturated fatty acid; PUFA, polyunsaturated fatty acid; O6/O3, ratio of linoleic and linolenic acid. Letters denote significant differences between storage weeks within the same cultivar samples (*ρ* < 0.001). Letters of the last two rows indicate significant differences between cultivar samples (*ρ* < 0.05). (n.d.) not detected.

**Table 3:** Standardized canonical discriminant function coefficients from discriminant analysis using stepwise method.

Volatile Compound	Function 1	Function 2
2-methylpropanal	0.634	0.872
Pentanal	2.199	6.332
Hexanal	−3.435	−9.647
(E)-2-pentenal	1.807	−3.127
3-octanone	3.205	0.49
Octanal	12.014	−5.081
(Z)-2-penten-1-ol	4.284	1.278
Hexanol	−2.248	0.14
(E)-2-octenal	2.8	−6.16
1-octen-3-ol	−16.793	13.871
Benzaldehyde	−1.068	0.667
(E,E)-2,4-nonadienal	−2.105	0.867
Hexanoic acid	1.294	−0.491

**Table 4:** Classification results for discriminant analysis model using ‘leave-one-out classification’ as cross-validation method for hierarchical cluster analysis (HCA) groups.

Groups	Predicted Group Membership			Total
	A1	A2	B	
A1	30 (100%)	0	0	30 (100%)
A2	2 (7.4%)	25 (92.6%)	0	27 (100%)
B	0	0	9 (100%)	9 (100%)

## Data Availability

The data presented in this study are available in the article and the Supplementary Materials.

## Supplementary Materials

The following are available online at https://www.mdpi.com/2304-8158/10/2/329/s1, Table S1: Selected fatty acids (% of total fatty acids detected) in the oil extracted from Chandler and Howard extracted at eleven time points for 28 weeks storage time; Table S2: Kernel volatile compounds (mean ± standard deviation; n from Chandler at 11 sampling times during 28 weeks of storage; Table S3: Kernel volatile compounds (mean ± standard deviation; n = 3) from Howard at 11 sampling times during 28 weeks of storage; Table S4: Oil volatile compounds (mean ± standard deviation; n = 3) from Chandler at 11 sampling times during 28 weeks of storage; Table S5: Oil volatile compounds (mean ± standard deviation; n = 3) from Howard at 11 sampling times during 28 weeks of storage.
