# Supplementary material for: Walnut (Juglans regia L.) Volatile Compounds Indicate Kernel and Oil Oxidation
Source: Foods. 2021 Feb 4;10(2):329. doi: 10.3390/foods10020329 (PMC7913853; doi:10.3390/foods10020329)
Supplement: Supplementary file 1 [file foods-10-00329-s001.pdf]

Supplementary Material

Figure S1. Chromatogram obtained from walnut (a) kernel and (b) oil volatile analysis.

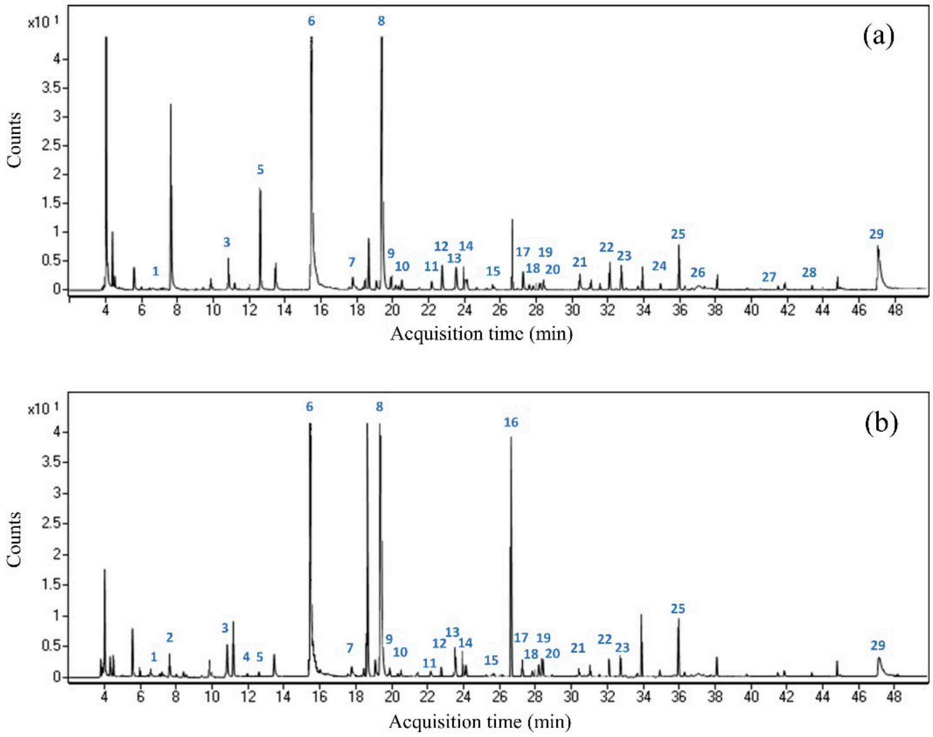

Table S1. Identified volatile compounds and retention times (Rt).

#	Rt (min)	Volatile compound
1	7.1	2-methylpropanal
2	7.6	Ethyl acetate
3	10.8	Pentanal
4	12.4	3-carene
5	12.6	1-penten-3-one
6	15.4	Hexanal
7	17.9	*(E)*-2-pentenal
8	19.4	1-penten-3-ol
9	19.9	2-heptanone
10	20.5	Heptanal
11	22.2	2-hexenal
12	22.7	2-pentyl-furan
13	23.5	1-pentanol
14	23.9	3-octanone
15	25.6	Octanal
16	26.6	1-octen-3-one
17	26.9	*(Z)*-2-penten-1-ol
18	27.3	*(E)*-2-heptenal
19	27.8	6-methyl-5-hepten-2-one
20	28.4	1-hexanol
21	30.4	Nonanal
22	32.1	*(E)*-2-octenal
23	32.7	1-octen-3-ol
24	34.9	*(E,E)*-2,4-heptadienal
25	36.3	Benzaldehyde
26	37.4	1-octanol
27	41	*(E)*-2-decenal
28	43.2	*(E,E)*-2,4-nonadienal
29	47.2	Hexanoic acid

Table S2. Selected fatty acids (% of total fatty acids detected) in the oil extracted from Chandler and Howard extracted at eleven time points for 28 weeks storage time.

Cultivar	Storage(weeks)	C16:0	C16:1	C17:0	C17:1	C18:0	C18:1	C18:2	C18:3	C22:0	C22:1
Chandler	0	5.1 ±0.80	5.8 ±0.10	0.1 ±0.00	0.1 ±0.00	2.1 ±0.31	14.6 ±0.18	57.3 ±0.65	14.6 ±0.16	0.1 ±0.01	0.2 ±0.00
	1	5.0 ±0.33	5.6 ±0.02	0.1 ±0.01	0.1 ±0.00	2.2 ±0.00	14.0 ±0.46	58.2 ±0.09	14.6 ±0.05	0.1 ±0.00	0.2 ±0.00
	2	4.3 ±0.01	5.6 ±0.00	0.1 ±0.00	0.1 ±0.00	2.2 ±0.00	14.7 ±0.01	58.1 ±0.05	14.6 ±0.02	0.1 ±0.00	0.2 ±0.00
	3	3.9 ±0.31	5.6 ±0.01	0.1 ±0.00	0.1 ±0.00	2.2 ±0.00	14.8 ±0.07	58.4 ±0.18	14.6 ±0.06	0.1 ±0.00	0.2 ±0.00
	4	4.6 ±0.38	6.2 ±0.08	0.1 ±0.00	0.1 ±0.01	2.2 ±0.48	14.7 ±0.18	57.4 ±0.47	14.4 ±0.12	0.1 ±0.00	0.2 ±0.00
	8	3.8 ±0.18	5.8 ±0.02	0.1 ±0.00	0.1 ±0.01	2.0 ±0.43	14.8 ±0.07	58.5 ±0.41	14.7 ±0.12	0.1 ±0.00	0.2 ±0.00
	12	3.5 ±0.42	5.7 ±0.03	0.1 ±0.00	0.1 ±0.02	2.3 ±0.02	14.9 ±0.06	58.5 ±0.33	14.7 ±0.10	0.1 ±0.00	0.2 ±0.00
	16	5.6 ±1.66	5.5 ±0.12	0.1 ±0.00	0.1 ±0.01	2.2 ±0.05	14.6 ±0.26	57.2 ±0.97	14.4 ±0.26	0.1 ±0.00	0.2 ±0.00
	20	4.2 ±1.10	5.8 ±0.01	0.1 ±0.00	0.1 ±0.00	2.0 ±0.38	15.2 ±0.13	57.6 ±0.47	14.8 ±0.08	0.1 ±0.01	0.2 ±0.00
	24	4.7 ±0.22	5.6 ±0.01	0.1 ±0.00	0.1 ±0.01	2.2 ±0.00	14.7 ±0.10	58 .1±0.17	14.4 ±0.04	0.1 ±0.00	0.2 ±0.00
	28	3.8 ±0.05	5.8 ±0.00	0.1 ±0.00	0.1 ±0.01	2.4 ±0.00	14.9 ±0.01	58.0 ±0.05	14.7 ±0.01	0.1 ±0.00	0.2 ±0.00
Howard	0	7.9 ±0.90	6.0 ±0.14	0.1 ±0.01	0.1 ±0.00	2.2 ±0.05	12.3 ±0.75	57.6 ±0.33	13.7 ±0.29	0.1 ±0.00	0.2 ±0.00
	1	3.6 ±0.10	6.2 ±0.03	0.1 ±0.00	0.1 ±0.00	2.3 ±0.01	13.9 ±0.01	59.1 ±0.10	14.6 ±0.02	0.1 ±0.00	0.2 ±0.00
	2	3.7 ±0.21	6.3 ±0.01	0.1 ±0.00	0.1 ±0.01	2.3 ±0.00	13.8 ±0.04	58.9 ±0.12	14.6 ±0.04	0.1 ±0.00	0.2 ±0.00
	3	2.0 ±0.35	6.5 ±0.09	0.1 ±0.00	0.1±0.01	2.1 ±0.40	13.9 ±0.09	60.1 ±0.43	15.0 ±0.13	0.1 ±0.00	0.2 ±0.00
	4	8.1 ±0.26	6.2 ±0.06	0.1 ±0.00	0.1±0.00	2.3 ±0.02	12.8 ±0.18	56.4 ±0.20	13.8 ±0.2	0.1 ±0.00	0.2 ±0.00
	8	5.0 ±0.08	6.4 ±0.06	0.1 ±0.00	0.1 ±0.00	2.3 ±0.04	13.6 ±0.16	57.9 ±0.10	14.4 ±0.05	0.1 ±0.00	0.2 ±0.00
	12	5.9 ±0.23	6.3 ±0.02	0.1 ±0.00	0.1 ±0.00	2.3 ±0.01	13.2 ±0.03	57.6 ±0.14	14.2 ±0.03	0.1 ±0.00	0.2 ±0.00
	16	3.6 ±0.14	6.0 ±0.05	0.1 ±0.01	0.1 ±0.00	2.5 ±0.01	14.1 ±0.06	59.5 ±0.21	14.0 ±0.06	0.1 ±0.00	0.2 ±0.00
	20	4.4 ±0.19	6.0 ±0.00	0.1 ±0.00	0.1 ±0.01	2.5 ±0.01	14.0 ±0.05	58.8 ±0.13	13.9 ±0.03	0.1 ±0.00	0.2 ±0.00
	24	3.6 ±0.17	6.0 ±0.01	0.1 ±0.00	0.1 ±0.00	2.5 ±0.00	14.4 ±0.02	59.1 ±0.11	14.1 ±0.03	0.1 ±0.00	0.2 ±0.00
	28	2.7 ±0.64	6.2 ±0.02	0.1 ±0.02	0.1 ±0.01	2.5 ±0.00	14.1 ±0.38	59.8 ±0.26	14.2 ±0.06	0.1 ±0.00	0.2 ±0.00
Chandler		4.4 ±0.81	5.7 ±0.18b	0.1 ±0.00b	0.1 ±0.01	2.2 ±0.21b	14.7 ±0.3a	57.9 ±0.55b	14.6 ±0.16a	0.1 ±0.01	0.2 ±0.00a
Howard		4.6 ±1.96	6.2 ±0.19a	0.1 ±0.01a	0.1 ±0.01	2.3 ±0.17a	13.6 ±0.63b	58.6 ±1.1a	14.2 ±0.4b	0.1 ±0.00	0.2 ±0.01b

Palmitic (C16:0), palmitoleic (C16:1), (17:0), stearic (C18:0), oleic (C18:1), linoleic (C18:2), linolenic (C18:3), (C22:0) and (C22:1) fatty acid average ± standard deviation. Letters denote significant differences between cultivars ($P < 0.05$) on the last two rows.

Table S3. Kernel volatile compounds (mean ± standard deviation; n from Chandler at 11 sampling times during 28 weeks of storage.

**Cultivar**	**Chandler**										
**Storage (weeks)**	**0**	**1**	**2**	**3**	**4**	**8**	**12**	**16**	**20**	**24**	**28**
2-methylpropanal	4.916±1.289	4.877±0.335	5.868±0.489	6.762±1.153	6.621±1.609	18.13±0.887	30.51±0.424	54.85±1.914	74.24±6.225	104.2±6.080	82.88±7.112
Ethyl acetate	n.d	n.d.	n.d.	n.d.	n.d.	n.d.	n.d.	n.d.	n.d.	n.d.	n.d.
Pentanal	14.52±6.296	13.37±1.237	14.01±0.705	19.22±1.220	11.20±2.145	109.4±7.327	140.2±12.31	276.5±50.51	626.1±12.85	610.3±20.47	639.2±59.588
3-carene	n.d	n.d.	n.d.	n.d.	n.d.	n.d.	n.d.	n.d.	n.d.	n.d.	n.d.
1-penten-3-one	5.100±2.226	4.466±1.234	7.091±1.117	6.910±0.483	3.995±0.664	18.71±2.016	22.36±1.051	30.73±3.578	57.05±3.420	49.74±1.596	67.59±4.128
Hexanal	312.7±43.74	344.6±24.99	437.4±46.82	475.2±17.00	239.9±160.8	1915±119.7	2475±88.05	5014±724.5	13124±378.6	12412±404.2	13518±1139
(E)-2-pentenal	48.08±9.268	34.64±6.701	36.2±26.443	33.03±26.32	41.07±11.19	31.63±1.056	52.15±1.102	73.53±7.446	136.8±12.37	123.6±4.430	130.6±10.10
1-penten-3-ol	2497±36.95	2291±313.5	2779±433.2	2558±261.9	2647±196.5	226.4±3.893	322.3±4.004	467.0±27.43	703.8±64.51	616.0±50.51	576.1±94.79
2-heptanone	92.65±63.491	100.2±20.40	118.2±30.56	91.77±14.95	88.55±10.33	65.58±10.47	22.58±0.892	51.61±7.608	144.6±7.417	129.3±13.69	194.6±15.28
Heptanal	10.74±2.307	10.24±1.012	11.52±1.522	9.375±0.456	10.19±2.59	20.11±0.521	24.16±0.496	45.63±4.607	151.4±5.062	106.5±4.21	152.4±17.87
2-hexenal	3.234±0.835	3.191±0.305	7.481±0.569	5.145±0.442	1.820±0.688	11.19±0.409	17.49±0.619	44.37±4.458	121.3±4.344	115.1±2.481	102.6±8.173
2-pentyl-furan	40.28±7.280	48.93±11.27	305.5±24.93	33.06±2.933	37.92±13.99	54.90±0.952	67.67±0.853	140.5±9.365	993.1±41.11	409.6±10.07	582.0±63.14
1-pentanol	30.02±10.18	28.75±1.687	28.09±2.815	30.747±2.34	30.58±7.915	104.8±4.276	147.2±16.64	405.6±62.52	1035±40.26	1084±59.16	1132±111.7
3-octanone	1.646±0.438	1.424±0.230	1.692±0.123	2.005±0.156	3.442±1.160	4.256±0.066	4.763±0.185	8.502±0.197	22.06±0.167	16.82±0.176	24.21±1.408
Octanal	4.546±1.200	4.523±0.310	6.356±1.516	3.261±0.198	4.071±1.185	5.426±0.334	7.884±1.462	13.94±3.378	63.15±0.626	45.74±5.626	71.52±13.38
1-octen-3-one	n.d	n.d.	n.d.	n.d.	n.d.	n.d.	n.d.	n.d.	n.d.	n.d.	n.d.
(Z)-2-penten-1-ol	1.792±0.608	1.704±0.160	2.330±0.295	3.059±0.403	1.791±0.273	10.18±0.367	15.37±0.242	31.72±2.478	58.21±3.123	50.93±1.920	60.81±7.43
(E)-2-heptenal	8.420±1.264	9.516±0.597	15.17±2.128	17.28±0.986	8.314±1.924	44.52±1.529	74.34±2.394	121.3±6.866	290.3±7.285	244.8±1.095	333.5±35.27
6-methyl-5-hepten-2-one	13.27±1.594	13.24±1.469	17.24±3.233	17.36±0.852	15.56±2.626	14.75±0.241	15.73±1.123	30.79±1.737	49.84±2.337	41.70±2.537	42.96±3.200
1-hexanol	38.85±5.932	49.06±3.396	35.45±2.425	48.71±3.033	79.39±24.49	64.76±0.528	99.11±0.634	218.9±6.990	432.9±21.96	408.4±11.88	537.3±47.46
Nonanal	13.04±1.627	13.94±1.493	16.45±2.823	10.75±0.368	15.99±5.518	15.87±1.080	20.50±0.935	31.91±2.440	96.36±3.923	58.32±4.058	73.92±8.621
(E)-2-octenal	5.635±1.684	5.535±0.567	3.093±0.511	3.468±0.393	3.459±1.804	12.25±0.926	14.90±0.985	59.04±6.865	233.4±1.739	215.1±8.391	313.8±52.10
1-octen-3-ol	8.340±1.220	10.05±0.915	19.72±1.569	23.63±1.836	15.58±6.063	56.00±2.298	104.6±0.877	214.9±15.88	553.5±12.92	480.3±8.17	602.9±123.2
(E,E)-2,4-heptadienal	7.927±1.855	8.099±1.281	10.53±3.044	13.42±1.608	7.867±1.336	41.08±4.592	67.14±2.086	133.2±5.506	227.8±14.69	208.8±7.402	199.1±31.93
Benzaldehyde	32.48±23.26	19.72±0.912	22.95±2.079	15.10±0.945	19.03±3.418	22.13±1.445	25.49±0.638	35.63±0.622	75.56±6.391	44.57±1.618	59.85±22.46
1-octanol	3.176±0.268	3.525±0.430	4.918±0.527	2.693±0.079	4.507±1.709	2.956±0.533	3.935±1.648	8.057±2.378	27.62±4.600	30.65±3.996	31.48±9.784
(E)-2-decenal	0.153±0.070	0.190±0.079	0.154±0.075	0.258±0.216	0.088±0.065	0.740±0.381	2.231±0.107	3.724±2.811	2.195±0.147	2.242±0.363	4.461±1.469
(E,E)-2,4-nonadienal	0.508±0.069	0.528±0.022	0.443±0.122	0.635±0.024	0.437±0.245	1.792±0.089	3.130±0.144	8.766±0.692	24.84±1.536	29.72±1.381	35.73±8.829
Hexanoic acid	0.555±0.516	0.312±0.041	0.206±0.179	0.244±0.026	0.143±0.077	1.223±0.649	0.887±0.561	10.45±1.016	33.63±4.168	35.10±8.167	10.07±0.378
∑VOCs	3200±47.59	3027±371.2	3907±512.3	3431±311.5	3299±45.57	2875±120.2	3782±108.9	7536±939.5	19360±591.5	17675±458.5	19581±1758
Alcohols	2577±31.42	2381±316.0	2864±439.4	2664±268.8	2774±176.3	462.2±2.200	688.6±13.29	1338±104.1	2783±121.1	2640±123.0	2910±367.7
Aldehydes	453.8±103.3	452.6±33.88	564.1±47.56	597.0±27.61	350.5±161.0	2225±120.7	2925±97.28	5868±817.2	15146±432.8	14245±418.4	15618±1343

Ketones	112.6±60.04	119.3±22.69	144.2±34.66	118.0±16.13	111.5±10.65	103.3±12.58	65.44±1.170	121.6±12.61	273.5±10.41	237.6±15.22	329.4±17.69
Furans	40.28±7.280	48.93±11.27	305.5±24.93	33.06±2.933	37.92±13.99	54.90±0.952	67.67±0.853	140.5±9.365	993.1±41.11	409.6±10.07	582.1±63.14
Acids	0.555±0.516	0.312±0.041	0.206±0.179	0.244±0.026	0.143±0.077	1.223±0.649	0.887±0.561	10.44±1.016	33.64±4.168	35.10±8.167	10.07±0.378

Values of volatile compounds expressed in µg of internal standard per kg of sample. (n.d.) not detected.

Table S4. Kernel volatile compounds (mean ± standard deviation; n=3) from Howard at 11 sampling times during 28 weeks of storage.

**Cultivar**	**Howard**										
**Storage (weeks)**	**0**	**1**	**2**	**3**	**4**	**8**	**12**	**16**	**20**	**24**	**28**
2-methylpropanal	6.007±0.925	5.264±0.611	12.49±0.831	9.324±1.208	26.80±0.486	50.39±11.95	85.59±5.771	118.8±4.439	188.6±17.29	266.6±17.50	245.7±23.24
Ethyl acetate	n.d	n.d.	n.d.	n.d.	n.d.	n.d.	n.d.	n.d.	n.d.	n.d.	n.d.
Pentanal	19.93±3.358	17.05±0.791	197.7±11.42	207.86±6.24	596.6±35.63	865.6±204.7	1077±22.56	1285±52.27	2027±110.4	2459±43.30	2921±129.1
3-carene	n.d	n.d.	n.d.	n.d.	n.d.	n.d.	n.d.	n.d.	n.d.	n.d.	n.d.
1-penten-3-one	4.915±0.218	3.335±1.140	19.49±0.979	29.37±2.810	73.62±1.527	90.49±18.78	129.4±6.214	150.6±4.118	175.5±10.36	316.4±2.193	321.5±36.06
Hexanal	425.3±78.11	391.7±42.13	4722±322.9	3905±394.6	11423±1021	16805±3813	31156±990	28363±1655	55793±2640	52525±340	61470±2170
(E)-2-pentenal	22.72±1.592	19.71±2.866	27.85±1.241	27.85±1.289	100.2±8.182	194.9±54.38	153.6±4.553	528.7±16.06	494.2±23.67	1128±18.52	1309±67.01
1-penten-3-ol	2376±132.0	2304±111.7	236.5±6.888	311.8±9.928	692.9±23.78	1035±247.1	1387±56.07	1443±65.132	2687±140.5	2552±70.08	2809±151.2
2-heptanone	25.23±2.535	19.45±1.874	38.38±4.306	33.08±2.537	85.53±6.295	132.4±35.68	391.5±26.19	334.7±40.69	1303±57.06	1115±33.88	1650±66.54
Heptanal	11.84±0.317	10.29±0.454	35.07±0.820	27.38±4.161	84.14±9.979	148.3±43.53	373.2±39.66	379.6±48.97	1253±44.64	992.5±26.12	1331±60.34
2-hexenal	1.192±0.338	1.243±0.959	27.42±1.322	21.53±0.772	68.19±6.981	113.9±29.78	223.7±13.81	261.7±20.45	559.1±16.90	625.2±15.32	732.3±33.78
2-pentyl-furan	124.6±15.90	100.6±8.531	382.1±6.727	98.55±13.47	352.2±32.31	498.7±154.5	1474±141.1	1669±265.8	6628±324.1	4792±180.40	7164±668.3
1-pentanol	101.2±9.653	89.35±6.398	409.5±30.60	436.4±24.91	15154±98.59	2202±514.1	3564±112.4	3672±171.3	6152±286.2	5456±19.14	6013±197.0
3-octanone	5.686±0.692	5.368±0.599	8.603±0.462	8.101±0.373	21.00±1.763	27.31±6.700	69.21±7.040	62.61±5.726	223.2±10.76	165.4±10.98	208.4±22.57
Octanal	4.821±0.266	3.007±0.067	10.622±0.75	6.349±1.502	22.04±2.470	42.09±14.38	154.9±19.84	144.0±17.65	713.0±31.97	513.8±12.26	756.4±58.24
1-octen-3-one	n.d	n.d.	n.d.	n.d.	n.d.	n.d.	n.d.	n.d.	n.d.	n.d.	n.d.
(Z)-2-penten-1-ol	1.142±0.427	1.014±0.373	15.31±0.255	19.54±0.836	59.85±5.636	109.3±26.67	196.1±15.00	187.4±11.99	431.9±12.86	390.9±8.115	337.3±17.60
(E)-2-heptenal	4.370±1.462	2.337±0.231	68.36±2.450	78.44±10.15	261.5±24.18	375.2±90.77	810.6±72.39	975.8±114.6	2437±99.73	2327±85.89	3238±246.7
6-methyl-5-hepten-2-one	18.77±0.543	18.006±1.89	18.04±0.612	15.02±2.013	21.88±2.347	30.33±6.677	32.08±1.446	66.09±3.416	125.0±3.733	77.24±0.210	64.91±9.665
1-hexanol	512.7±20.49	490.4±17.85	152.9±0.675	126.4±15.37	292.4±43.43	409.7±103.9	691.8±24.08	2412±264.2	4717±374.5	1402±16.32	1872±99.71
Nonanal	17.07±1.134	13.60±0.595	19.91±0.190	17.53±4.765	29.11±3.482	49.62±15.05	151.6±17.27	144.6±19.48	615.1±51.13	426.8±23.89	576.3±76.93
(E)-2-octenal	4.781±2.261	2.046±0.304	53.67±5.801	46.52±10.00	267.5±27.22	381.9±97.56	917.8±103.6	1124±215.8	3256±198.0	2712±155.9	3874±409.8
1-octen-3-ol	29.90±4.407	23.20±1.706	115.7±8.169	106.5±22.37	429.1±36.95	657.3±156.3	1656±186.2	1647±221.1	4819±221.7	3861±112.1	5555±447.8
(E,E)-2,4-heptadienal	4.700±0.600	3.414±0.179	25.33±1.204	28.99±9.498	89.88±13.19	164.4±37.20	430.2±44.63	424.5±21.06	1014±23.33	985.6±4.034	1294±107.9
Benzaldehyde	18.40±3.197	12.40±0.906	25.59±3.591	14.73±2.808	21.24±3.465	31.45±6.780	48.66±0.865	61.67±5.322	172.7±6.869	121.9±5.284	124.3±15.58
1-octanol	5.766±0.801	3.766±0.268	8.302±2.870	4.187±1.694	19.93±4.244	50.41±13.35	119.4±15.32	82.60±18.47	409.0±44.60	316.7±68.76	510.8±61.08
(E)-2-decenal	0.279±0.098	0.173±0.154	0.406±0.070	0.216±0.034	1.504±0.324	4.233±2.161	15.71±3.552	10.47±1.495	52.86±8.471	45.44±4.075	60.54±11.44
(E,E)-2,4-nonadienal	1.540±1.158	0.633±0.171	2.993±0.471	2.596±0.542	15.17±1.964	31.30±11.85	113.2±23.07	80.99±10.74	343.0±48.76	313.5±28.22	418.8±55.70
Hexanoic acid	0.440±0.219	3.508±1.063	3.786±1.344	0.784±0.883	8.649±3.637	21.57±10.26	144.2±72.59	84.47±20.84	763.3±105.1	690.6±89.61	857.3±113.8
∑VOCs	3749±28.82	3545±160.1	6638±395.0	5585±520.0	16580±1395	24525±5625	45570±1869	45719±3078	97358±4436	86584±446.7	105723±5022
Alcohols	3021±99.54	2908±133.4	930.1±42.79	1001±65.19	2989±202.2	4414±1026	7496±368.0	9364±627.9	18807±868.3	13664±47.41	16589±830.0
Aldehydes	522.7±86.45	469.7±38.55	5200±342.9	4377±434.5	12969±1148	19192±4379	35535±1313	33751±2170	68353±3115	64964±24.29	77751±3302
Ketones	54.60±3.978	46.16±5.188	84.53±3.246	85.57±4.848	202.1±10.19	280.5±66.94	622.3±37.38	614.1±51.38	1827±81.23	1674±47.26	2245±111.0
Furans	124.6±15.90	100.63±8.531	382.1±6.727	98.55±13.47	352.2±32.31	498.7±154.5	1474±141.0	1669±265.8	6628±324.1	4792±180.4	7164±668.3
Acids	0.440±0.219	3.508±1.063	3.786±1.344	0.784±0.883	8.649±3.637	21.57±10.26	144.2±72.59	84.47±20.84	763.3±105.1	690.6±89.61	857.3±113.8

Values of volatile compounds expressed in µg of internal standard per kg of sample. (n.d.) not detected.

Table S5. Oil volatile compounds (mean ± standard deviation; n=3) from Chandler at 11 sampling times during 28 weeks of storage.

**Cultivar**	**Chandler**										
**Storage (weeks)**	**0**	**1**	**2**	**3**	**4**	**8**	**12**	**16**	**20**	**24**	**28**
2-methylpropanal	3.746±0.521	3.131±0.347	3.627±0.770	8.574±1.411	3.914±1.383	45.14±56.62	5.996±1.169	9.573±0.823	22.01±0.961	36.79±1.837	58.76±13.38
Ethyl Acetate	573.7±54.81	81.79±1.920	52.55±8.018	81.21±0.084	298.5±12.02	45.313±1.15	151.3±10.11	146.8±14.72	95.77±7.760	35.09±0.770	73.86±36.31
Pentanal	11.39±1.109	11.81±0.621	21.17±2.767	56.06±2.954	16.62±0.110	37.42±2.884	30.22±0.702	56.73±9.390	180.2±19.17	274.8±9.535	520.6±112.5
3-Carene	11.66±1.032	8.234±0.107	8.175±0.877	7.896±0.208	5.962±0.102	7.329±0.609	5.785±0.445	3.604±0.620	4.697±0.510	5.159±0.058	3.723±0.879
1-Penten-3-one	4.863±0.974	4.784±0.230	11.33±2.969	17.56±0.006	10.92±0.472	11.908±1.79	17.80±1.480	19.95±2.701	27.45±1.155	33.931±2.08	45.13±10.69
Hexanal	214.1±16.47	374.1±10.08	514.6±64.81	874.38±23.9	300.9±9.580	761.6±23.00	566.6±22.74	1044±137.7	3564.3±2.02	4615±37.48	10480±2322
(E)-2-Pentenal	18.38±2.912	14.93±0.514	18.44±2.237	6.240±0.281	7.370±8.111	11.78±10.27	13.42±0.870	10.23±1.384	30.81±0.806	48.52±2.441	137.6±29.03
1-Penten-3-ol	920.2±62.21	1055±11.51	521.1±54.10	99.10±3.025	65.01±6.682	117.5±3.966	174.3±13.52	192.7±17.56	351.8±10.48	446.9±13.45	479.3±117.8
2-Heptanone	9.940±1.393	13.68±1.859	14.77±1.243	13.65±0.093	11.59±0.762	13.01±1.056	9.020±1.242	15.65±1.844	38.17±2.999	54.60±1.133	158.2±31.99
Heptanal	3.904±0.303	5.058±0.308	5.382±0.769	7.880±0.364	2.984±0.304	12.51±0.829	6.816±0.531	9.843±1.785	30.15±1.655	35.91±1.747	153.8±35.08
2-Hexenal	0.311±0.160	3.456±0.212	1.494±0.140	3.252±0.239	1.224±0.221	6.205±0.054	4.454±0.065	7.253±0.736	24.25±1.166	28.00±0.177	90.15±18.92
2-pentyl-Furan	10.62±0.927	15.37±0.477	13.04±1.606	24.05±0.581	8.401±0.751	45.16±0.182	34.87±1.763	50.73±5.169	115.8±0.182	149.2±1.432	956.9±220.1
1-Pentanol	12.95±5.305	15.55±0.671	21.47±3.617	86.42±3.209	16.31±1.817	60.88±1.532	45.62±3.708	82.84±11.70	257.4±4.285	316.299±1.8	584.9±126.2
3-Octanone	0.455±0.117	1.105±0.160	1.745±0.257	2.884±0.160	1.378±0.135	3.866±0.070	3.979±0.123	4.913±0.655	7.387±1.030	9.107±0.564	14.42±3.582
Octanal	2.842±1.458	2.126±0.008	1.329±0.778	2.812±0.008	1.075±0.048	4.391±0.170	2.211±0.086	2.778±0.232	9.145±2.952	13.58±0.462	62.33±15.18
1-octen-3-one	4909±845.3	5182±820.8	4148±721.1	4252±1115	2672±598.8	5026±258.1	4401±93.53	3110±511.9	5427±96.33	3666±1371	5091±1692
(Z)-2-Penten-1-ol	0.697±0.159	1.376±0.321	1.646±0.305	3.108±0.063	1.741±0.144	6.932±0.023	6.854±0.347	9.201±1.544	18.37±1.356	25.04±0.008	46.60±10.92
(E)-2-Heptenal	2.203±0.381	5.696±0.150	8.778±1.443	17.43±0.633	9.973±1.120	23.05±1.234	24.564±1.41	28.322±3.38	75.02±2.101	102.5±1.139	240.1±50.48
6-methyl-5-Hepten-2-one	4.524±0.347	5.653±0.219	4.856±0.897	8.211±0.914	4.569±0.521	14.32±0.558	13.12±0.809	17.65±1.904	20.31±1.968	18.74±0.605	32.17±7.549
1-Hexanol	11.36±2.121	15.71±0.099	21.26±2.398	20.38±0.618	12.99±1.175	67.61±2.195	37.23±2.147	72.07±9.528	74.72±8.804	98.98±1.400	250.1±54.24
Nonanal	7.220±3.133	7.500±0.874	6.290±1.254	6.340±0.158	3.301±0.404	11.55±0.178	7.430±0.886	6.920±0.949	14.44±0.709	17.40±0.363	89.94±19.81
(E)-2-Octenal	0.902±0.059	1.623±0.111	2.238±0.068	6.845±0.005	1.372±0.094	5.844±0.247	3.565±0.044	7.889±1.219	40.12±3.189	71.12±0.638	203.7±44.77
1-Octen-3-ol	5.200±0.118	8.920±0.088	12.89±1.345	22.20±0.567	11.20±1.240	40.17±0.302	36.71±2.451	55.45±7.691	128.6±6.827	191.2±3.528	421.1±95.41
(E,E)-2,4-Heptadienal	n.d	n.d.	n.d.	n.d.	n.d.	n.d.	n.d.	n.d.	n.d.	n.d.	n.d.
Benzaldehyde	13.281±3.192	11.54±0.430	11.203±1.05	11.714±0.16	7.476±0.628	18.86±0.304	14.51±0.602	14.115±2.29	23.29±5.145	20.92±0.569	49.33±11.77
1-Octanol	n.d	n.d.	n.d.	n.d.	n.d.	n.d.	n.d.	n.d.	n.d.	n.d.	n.d.
(E)-2-Decenal	n.d	n.d.	n.d.	n.d.	n.d.	n.d.	n.d.	n.d.	n.d.	n.d.	n.d.
(E,E)-2,4-Nonadienal	n.d	n.d.	n.d.	n.d.	n.d.	n.d.	n.d.	n.d.	n.d.	n.d.	n.d.
Hexanoic acid	14.169±7.273	19.62±0.994	27.23±6.285	62.98±12.06	12.10±1.590	45.97±4.067	33.12±2.687	77.36±15.47	357.95±41.78	965.5±66.42	2074±579.4
ΣVOCs	6769±823.25	6871±172.9	5455±1166	5704±638.4	3489±346.4	6444±23.81	5651±761.3	5058±44.07	10940±1471	11282±5000	22320±276.4
Alcohols	950.4±69.68	1097±10.71	578.3±646.4	231.2±7.360	107.2±10.97	293.1±7.410	300.7±22.17	412.2±48.04	830.9±10.79	1078±13.77	1782±402.3
Aldehydes	265.0±16.60	429.5±9.590	583.4±75.04	989.8±29.61	348.8±13.74	919.5±71.19	665.3±28.29	1183±155.9	3990±5.540	5244±48.59	12038±2657
Ketones	4929±847.4	5208±823.0	4181±726.5	4295±1116	2700±599.5	5069±261.49	4445±89.88	3169±519.1	5521±89.17	3783±1372	5341±1722
Terpenes	10.62±0.927	15.37±0.477	13.04±1.606	24.05±0.581	8.401±0.751	45.16±0.182	34.87±1.763	50.73±5.169	115.8±0.182	149.2±1.432	956.9±220.1
Furans	14.00±7.300	20.00±0.990	26.90±6.300	63.01±12.10	12.01±1.600	46.00±4.100	33.00±2.700	77.00±15.50	358.0±41.80	966.0±66.40	2075±579.4
Esters	3.746±0.521	3.131±0.347	3.627±0.770	8.574±1.411	3.914±1.383	45.14±56.62	5.996±1.169	9.573±0.823	22.01±0.961	36.79±1.837	58.76±13.38
Acid	573.7±54.81	81.79±1.920	52.55±8.018	81.21±0.084	298.5±12.02	45.313±1.15	151.3±10.11	146.8±14.72	95.77±7.760	35.09±0.770	73.86±36.31

Values of volatile compounds expressed in μg of internal standard per kg of sample. (n.d.) not detected.

Table S6. Oil volatile compounds (mean ± standard deviation; n=3) from Howard at 11 sampling times during 28 weeks of storage.

**Cultivar**	**Howard**										
**Storage (weeks)**	**0**	**1**	**2**	**3**	**4**	**8**	**12**	**16**	**20**	**24**	**28**
2-methylpropanal	1.711±0.122	4.671±0.035	12.30±0.108	12.42±0.964	38.88±2.526	44.22±2.400	62.85±15.14	73.61±4.588	148.1±2.813	170.6±0.903	173.5±5.615
Ethyl Acetate	98.86±7.834	43.11±0.059	36.858±0.09	27.23±5.203	11.45±0.025	79.74±1.593	11.13±0.203	9.342±0.440	89.12±2.032	2.225±0.344	21.67±2.686
Pentanal	11.74±1.411	37.47±0.728	102.9±3.646	117.1±0.014	402.2±3.643	496.0±3.759	681.4±169.6	854.9±20.91	1190±0.545	1290±19.98	1547±17.85
3-Carene	13.06±1.651	8.568±0.736	8.425±0.469	7.605±0.650	9.618±0.680	4.987±0.029	9.063±0.374	8.022±1.269	4.149±0.030	4.914±0.509	4.628±0.136
1-Penten-3-one	3.972±0.054	8.430±0.128	19.03±0.884	17.83±3.668	42.23±0.824	53.71±1.696	69.57±16.87	81.46±7.567	120.5±9.733	153.8±4.291	160.1±7.129
Hexanal	334.5±26.29	653.8±20.21	1831±28.58	2300±324.2	7196±159.3	9283±80.46	13502±3728	17948±341.8	23155±98.90	24873±42.79	31777±528.9
(E)-2-Pentenal	14.09±3.080	9.090±8.722	11.27±0.391	13.96±0.909	64.50±15.74	82.85±28.91	182.6±72.44	251.0±9.697	370.2±13.74	414.6±6.081	571.6±25.63
1-Penten-3-ol	1097±129.0	54.20±1.206	125.3±2.848	185.3±16.15	566.8±5.926	652.5±10.46	876.6±185.4	1105±5.095	1301±0.211	1559±14.50	1886±26.75
2-Heptanone	13.98±0.950	7.120±0.715	13.77±0.238	13.87±3.185	52.13±1.680	112.2±5.120	126.6±46.38	170.1±1.333	394.9±0.906	471.0±0.845	743.4±8.990
Heptanal	9.283±2.312	5.193±0.218	11.64±0.266	11.30±2.456	50.43±0.673	95.04±0.153	141.3±58.08	188.7±0.031	351.4±1.580	401.2±1.238	613.4±7.854
2-Hexenal	1.863±0.241	2.285±0.104	6.774±0.145	7.755±0.910	38.94±2.004	47.65±2.248	92.58±33.69	120.9±2.926	176.4±1.427	200.3±5.015	300.3±3.928
2-pentyl-Furan	32.30±3.204	16.31±1.343	33.51±0.437	32.88±1.910	221.9±3.436	386.2±22.38	649.5±253.9	943.6±13.95	1235±14.95	1693±13.50	3023±81.219
1-Pentanol	52.40±5.714	41.45±1.298	119.5±0.773	192.1±12.43	720.6±0.555	869.9±5.578	1304±349.5	1701±22.98	1850±12.02	2024±13.77	2460±42.951
3-Octanone	2.854±0.337	2.259±0.081	3.890±0.024	4.086±0.688	14.01±0.075	23.37±1.016	23.23±5.640	28.97±0.490	60.62±1.238	72.31±1.984	95.47±6.031
Octanal	4.142±0.558	1.412±0.329	2.825±0.581	2.352±0.365	15.25±0.665	34.31±0.441	42.71±16.49	59.87±2.211	148.7±0.094	183.1±4.216	263.0±20.46
1-octen-3-one	3748±479.3	3818±883.8	3313±848.5	4473±1146	4816±1105	2365±3168	5942±858.5	6574±201.4	8171±49.41	4289±2425	3622±5429
(Z)-2-Penten-1-ol	2.603±0.256	2.120±0.230	6.352±0.946	9.393±0.445	43.71±0.911	58.19±1.082	93.49±31.58	120.9±0.535	166.6±1.466	198.6±8.738	225.5±20.41
(E)-2-Heptenal	4.497±0.257	7.895±0.107	22.66±1.305	28.21±0.641	143.6±3.207	191.9±6.871	342.4±118.5	478.3±6.708	713.6±0.190	863.5±12.89	1264±41.31
6-methyl-5-Hepten-2-one	4.888±0.928	4.185±0.071	4.402±0.351	5.414±0.298	10.36±0.088	13.57±0.479	18.42±0.681	23.05±0.863	36.73±0.459	32.52±0.095	38.66±8.022
1-Hexanol	100.6±9.405	22.29±0.365	29.649±1.97	35.13±1.759	93.50±3.09	168.9±11.42	285.0±5.725	376.6±10.43	273.2±7.774	304.1±0.964	659.7±39.09
Nonanal	9.040±2.480	4.470±0.884	7.200±0.467	5.140±0.125	16.47±2.185	31.72±1.406	43.18±15.93	59.63±3.461	120.8±1.763	140.7±4.045	181.2±31.77
(E)-2-Octenal	4.690±0.700	2.727±0.336	11.41±0.447	14.93±0.755	138.8±3.605	203.6±14.69	340.3±118.1	482.67±11.95	760.8±2.605	936.2±11.77	1391±73.36
1-Octen-3-ol	14.27±1.794	11.82±0.151	31.16±0.904	39.04±1.057	223.5±6.146	365.7±22.66	534.8±184.5	748.1±16.236	1176±10.39	1437±15.66	2138±101.4
(E,E)-2,4-Heptadienal	n.d	n.d.	n.d.	n.d.	n.d.	n.d.	n.d.	n.d.	n.d.	n.d.	n.d.
Benzaldehyde	13.97±1.28	6.778±0.422	9.145±0.354	8.012±0.336	17.541±2.04	25.35±2.049	36.76±1.777	21.04±1.639	57.81±3.366	55.26±0.767	62.68±8.454
1-Octanol	n.d	n.d.	n.d.	n.d.	n.d.	n.d.	n.d.	n.d.	n.d.	n.d.	n.d.
(E)-2-Decenal	n.d	n.d.	n.d.	n.d.	n.d.	n.d.	n.d.	n.d.	n.d.	n.d.	n.d.
(E,E)-2,4-Nonadienal	n.d	n.d.	n.d.	n.d.	n.d.	n.d.	n.d.	n.d.	n.d.	n.d.	n.d.
Hexanoic acid	47.68±3.425	28.04±2.434	88.35±2.035	147.0±9.081	1303±4.060	3123±294.5	6019±287.1	2092±150.8	11621±392.4	14786±28.81	23182±865.6
ΣVOCs	5643±864.9	4804±830.3	5864±1487	7711±910.5	16252±2684	18815±6232	31433±97.67	34524±185.9	53699±5468	56559±5131	76409±0.110
Alcohols	1267±146.1	131.9±0.110	312.1±1.750	461.0±27.44	1648.1±4.78	2115±28.12	3094±746.1	4053±23.16	4768.7±28.52	5523±53.64	7371±188.8
Aldehydes	395.6±37.21	729.1±13.30	2020±22.41	2513±318.8	8105±192.2	10510±140.4	15431±4345	20518±404.3	27137.2±122.18	29474±3.09	38082±552.2
Ketones	3773±477.2	3840±883.2	3355±847.8	4514±1139	4934±1108	2568±3170	6180±920.5	6878±192.9	8784.23±39.553	5018±2426	4660±5416
Terpenes	32.30±3.204	16.31±1.343	33.51±0.437	32.88±1.910	221.9±3.436	386.2±22.38	649.5±253.9	943.6±13.95	1235.69±14.959	1693±13.50	3023±81.21
Furans	48.00±3.400	28.00±2.400	88.00±2.00	147.0±9.100	1303±4.100	3124±294.6	6020±287.2	2093±150.9	11622±392.5	14787±28.80	23182±865.7
Esters	1.711±0.122	4.671±0.035	12.30±0.108	12.42±0.964	38.88±2.526	44.22±2.400	62.85±15.14	73.61±4.588	148.1±2.813	170.6±0.903	173.5±5.615
Acid	98.86±7.834	43.11±0.059	36.858±0.09	27.23±5.203	11.45±0.025	79.74±1.593	11.13±0.203	9.342±0.440	89.12±2.032	2.225±0.344	21.67±2.686

Values of volatile compounds expressed in μg of internal standard per kg of sample. (n.d.) not detected.
